# Finite Element Analysis of Evolut Transcatheter Heart Valves: Effects of Aortic Geometries and Valve Sizes on Post-TAVI Wall Stresses and Deformations

**DOI:** 10.3390/jcm14030850

**Published:** 2025-01-27

**Authors:** Onur Mutlu, Noaman Mazhar, Murat Saribay, Mehmet Metin Yavuz, Deniz Ozturk, Abdel Naser Ghareeb, Abdulrahman Alnabti, Huseyin Cagatay Yalcin

**Affiliations:** 1 Biomedical Research Center, QU Health, Qatar University, Doha 2713, Qatar; onurmutlu94@gmail.com (O.M.); noaman.mazhar@qu.edu.qa (N.M.); 2 Mechanical Engineering Department, Istanbul Bilgi University, Istanbul 34050, Turkey; murat.saribay@bilgi.edu.tr; 3 Mechanical Engineering Department, Middle East Technical University, Ankara 06800, Turkey; ymetin@metu.edu.tr; 4 Medividia, 3020 Herent, Belgium; 5 Heart Hospital, Hamad Medical Corporation, Doha 3050, Qatar; nasergharib@yahoo.com; 6 Faculty of Medicine, Al Azhar University, Cairo 11884, Egypt; 7 Department of Biomedical Science, College of Health Sciences, QU Health, Qatar University, Doha 2713, Qatar; 8 Department of Mechanical and Industrial Engineering, Qatar University, Doha 2713, Qatar

**Keywords:** transcatheter aortic valve implantation, finite element analysis, aortic stenosis, Evolut-R, contact pressure, von Mises stresses, ABAQUS

## Abstract

**Background/Objectives:** For transcatheter aortic valve implantation (TAVI) therapy, a catheter-guided crimped valve is deployed into the aortic root. Valve types such as Edwards balloon-expandable valves and Medtronic self-expandable valves come in different sizes and are chosen based on patient-specific aortic anatomy, including aortic root diameter measurement. Complications may arise due to variations in anatomical characteristics and the implantation procedure, making pre-implantation assessment important for predicting complications. **Methods:** Computational modeling, particularly finite element analysis (FEA), has become popular for assessing wall stresses and deformations in TAVI. In this study, a finite element model including the aorta, native leaflets, and TAVI device was used to simulate procedures and assess patient-specific wall stresses and deformations. **Results:** Using the Medtronic Evolut R valve, we simulated TAVI for 14 patients to analyze the effects of geometrical variations on structural stresses. Virtual TAVIs with different valve sizes were also simulated to study the influence of TAV size on stresses. Our results show that variations in aortic wall geometries and TAV sizes significantly influence wall stresses and deformations. **Conclusions:** Our study is one of the first comprehensive FEA investigations of aortic geometrical variations and valve sizes on post-TAVI stresses, demonstrating the non-linear relationship between aortic dimensions, TAV sizes, and wall stresses.

## 1. Introduction

Among the most critical cardiovascular diseases (CVDs) are diseases related to the heart valves, particularly the aortic valve (AV). Aortic stenosis (AS) is characterized by the narrowing of the AV’s fully opened state during the systolic phase. This defect can either be congenital or develop later in life [1]. AS restricts the opening of the AV during ventricular systole, obstructing forward flow and causing pressure overload in the left ventricle. Moreover, diseased AV leaflets cannot completely close during ventricular diastole, causing regurgitation of blood flow to the left ventricle. While surgical valve repair is feasible for a severely damaged or non-functioning AV, the use of a prosthetic heart valve is a primary treatment option for the significant majority of patients [2].

Prosthetic AVs are grouped into mechanical and bioprosthetic heart valves (MHVs and BHVs, respectively). MHVs are composed of pyrolytic carbon and metal alloys, whereas BHVs are constructed from biologically sourced, exogenously cross-linked, soft collagenous tissue to form leaflet biomaterials, frequently sutured to a rigid stent [3]. Conventionally, MHVs or BHVs are surgically implanted via invasive open-heart surgery. However, transcatheter aortic valve implantation (TAVI) was introduced two decades ago as a minimally invasive alternative for the implantation of new-generation BHVs [4]. Here, a stented BHV is implanted into the aortic root with degenerative native leaflets. Placement trials of transcatheter aortic valves (TAVs) have demonstrated the superior effectiveness of TAVI in reducing short- and medium-term mortality. This has resulted in the establishment of TAVI as a new revolutionary treatment in the last decade, with a growing emphasis on its safety and efficacy [5]. Currently, there are two categories of TAVs in clinical practice: balloon-expandable valves and self-expandable valves. Balloon-expandable valves employ a stent composed of elastoplastic metal, while self-expandable valves are furnished with a nitinol super-elastic alloy [6].

The Medtronic CoreValve and Evolut-R are self-expanding TAVs. They have a tubular form skirt inflow and three flexible leaflets of porcine pericardium sutured onto a stent. The laser-cut stent consists of diamond-shaped cells with varying patterns along the axis. The three available sizes of CoreValve are Size 26, 29, and 34. These sizes are recommended for aortic annulus diameters of 20–23 mm, 23–26 mm, and 26–29 mm, respectively. The Evolut-R is offered in four sizes. Sizes 23, 26, 29, and 34 are recommended for aortic annulus diameters of 18 to 20 mm, 20 to 23 mm, 23 to 26 mm, and 26 to 30 mm, respectively. For a successful TAVI, the implanted valve should attach to the aortic wall well while not causing excessive mechanical stress on the annulus from the stent. A small valve implant can result in valve migration and paravalvular leakage, whereas a large valve implant can cause conduction problems and annular rupture [7,8]. Hence a reliable patient-specific mechanical assessment for the interaction of the TAV with aortic wall is needed that may be used for prediction of possible complications at a pre-operative stage.

Finite element analysis (FEA) is a very useful approach for the virtual surgery of TAVI in a patient-specific manner for mechanical assessment of TAV attachment on the aortic root. This technique can be used to assess different TAVs for a specific patient to identify the optimal valve with improved outcomes. Several mechanical stress parameters such as contact pressure from TAV stent on the aortic wall and von Mises stresses on the aortic wall can be calculated with this analysis [9,10]. These stresses should not be too low so that the TAV stent attaches properly on the wall, but also should not be too high, so as to not induce conduction abnormalities [11,12]. Using FEA virtual implantation of multiple TAV sizes simultaneously enables the calculation of the mechanical stresses on the patient’s aortic wall and native leaflets of these devices, allowing researchers to optimize TAVs [13,14,15]. Some recent FEA studies have focused on the determination of TAV stent contact areas on walls to assess anchoring [16,17], while others have shown interest in the computation of contact pressures on the aortic root to assess the risk of conduction problems [18,19] and structural stresses on the leaflets to assess the risk of tissue degeneration [20,21,22] for different valve designs and different implant depth positioning.

Since there are multiple-size TAVs for different brands, it is preferable to compare different-size valves in a patient-specific manner before TAVI. Smaller valves are expected to prevent conduction anomalies but may migrate, whereas larger valves can attach well but may cause conduction problems due to excessive pressure. In this study, retrospective data for patients enrolled in the Hamad Heart Hospital TAVI program were included for a comprehensive FEA examination of TAVI. Medtronic Evolut-R implanted cases were investigated since this valve type has been the most commonly used valve in the hospital. By comparing the results of patients who had the same type and size of valves implanted, we aimed to investigate the influence of patient-specific anatomical aortic geometrical variations on TAVI wall stresses. For each patient, in addition to the actually implanted valve simulations, we also virtually implanted a smaller valve and a larger valve to study the effect of valve size on aortic wall mechanics in a patient-specific manner. Our results showed the extreme importance of aortic geometrical variations for post-TAVI stresses, as well as the non-linear relation of aortic dimensions, TAV sizes, and post-TAVI wall stresses.

## 2. Materials and Methods

An FEA requires a set of inputs before proceeding with the solver step. These inputs include geometry, material properties, meshing, and other FEA information such as boundary, load, and contact conditions. In this section, the input preparation in a TAVI FE simulation will be explained in detail.

### 2.1. Generation of Model Geometries

To simulate a TAVI procedure with FEA, it is necessary to accurately model and assemble several components. To perform an FE simulation of the Evolut-R TAV, it is necessary to obtain the geometries of the aorta, native leaflets, stent, skirt, and prosthetic leaflets. This chapter provides an overview of the process of obtaining patient-specific geometries as a first step. Furthermore, the parametric modeling of native leaflets and the step-by-step modeling of the Evolut-R TAV are addressed.

#### 2.1.1. Extended Aortic Root and Descending Aorta Geometry Generation

Computed tomography (CT) images of 14 patients who underwent TAVI treatment at the Hamad Medical Corporation—Heart Hospital (HMC-HH) were included in the study. Both the collection of CT images and further analysis were carried out under IRB ethical approval obtained from HMC–HH (MRC-03-21-461). The study population comprised 10 male patients and 4 female patients. The average ages were 79 and 72 for male and female patients, respectively. The youngest and oldest patients in the male group were 60 and 93 years old, respectively. On the other hand, the youngest and oldest female patients were 67 and 81 years old, respectively. Main characteristics of the study cohort is summarized in Table 1.

**Figure 1:**
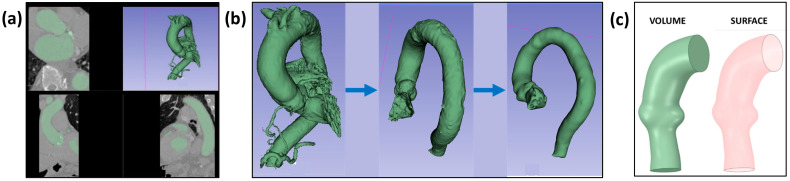
**Aorta geometry generation** (**a**) Patient-specific CT images, (**b**) 3D geometry segmentation using 3D Slicer software, and (**c**) final volume and surface geometries for FEA.

Among the included cases, 7 were implemented with a 26 mm valve size, 5 with a 29 mm valve size, and 2 with a 34 mm valve size. Obtained CT images (Figure 1a) were segmented (Figure 1b) using the open-source 3D Slicer (version 5.2.1, Slicer community, www.slicer.org) software. After that, aorta geometries in “.stl” format were re-meshed and reconstructed using the Mesh Mixer (version 3.5, Autodesk, Inc., San Rafael, CA, USA) software. Finally, the patient geometries in the “.stl” format were reconstructed again to obtain a combination of the extended aortic root and ascending aorta, then were converted to solid and surface geometries (Figure 1c) suitable for FEA analysis using SpaceClaim (version 19.2, Ansys, Inc., Canonsburg, PA, USA) software. Although the aorta was exported and used as a surface geometry, a uniform thickness value of 2 mm [23,24,25,26] was assigned by using the Abaqus (version 2023; Dassault Systèmes Simulia Corp., Johnston, RI, USA) section assignment manager to have a final volume geometry.

#### 2.1.2. Native Leaflet Geometry Generation

**Figure 2:**
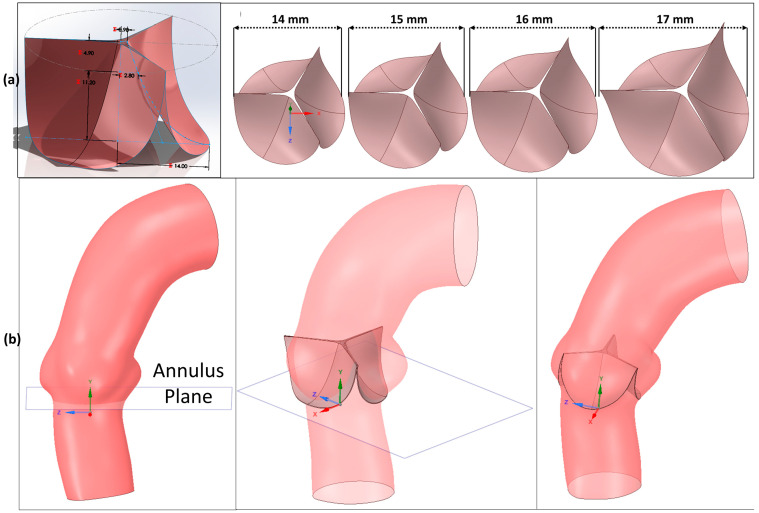
(**a**) **The parametric native valve geometry design:** Geometrical parameters (mm) of the native leaflets taken from the literature [28] are indicated with the red color, and the generated parametric native leaflets in different annulus diameters (14 mm, 15 mm, 16 mm, and 17 mm, respectively). (**b**) **The placement process of the parametric native valve geometry into the aortic root**.

In general, it is very difficult to segment the aortic leaflets from CT images due to their extremely thin structures and variable wall thicknesses. Therefore, the echocardiography technique is commonly used to accurately extract the shape and position of the aortic valve leaflets [21,22]. However, in the absence of echo images, the native leaflet is modeled using AV-related anatomical measurements [23,25,27]. In the current case, a parametric native leaflet modeling approach was adapted for all patient-specific cases due to the absence of echo images in most patients. Here, the parametric native leaflet geometry shown in Figure 2a was generated based on the information given in the literature. In this approach, valve size parameters depended on the annulus radius for each patient. Hence, four different parametric native valve geometries were designed using Solid Works (version 2024; Dassault Systèmes SolidWorks Corporation, Waltham, MA, USA), using an annulus radius of 14 mm, 15 mm, 16 mm, and 17 mm, respectively. A uniform leaflet thickness of 0.5 mm was implemented, similar to previous studies [21,22,23,24,25,26]. For each studied case, a slightly larger size of the parametric valve leaflets’ annular size was chosen according to the diameter of the aortic annulus. For placing the native leaflets, first, a plane was added to the ventriculo-aortic junction (Figure 2b), starting from the lower part of the sinus of Valsalva and intersecting the aortic annulus; then, the sinus of Valsalva was aligned with the midline of the leaflet of the parametric valve. To obtain the final shape, the parametric leaflet sections located outside the aorta were trimmed using the surface of the aorta.

#### 2.1.3. TAV Geometry Generation

**Figure 3:**
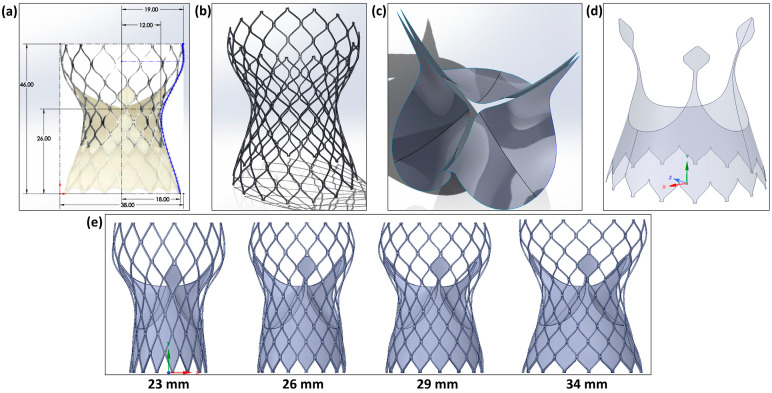
**The 23 mm, 26 mm, 29 mm, and 34 mm Medtronic Evolut-R TAV design steps**. (**a**) Surface spline created from the calibrated catalog image using the device dimensions, (**b**) stent, (**c**) prosthetic leaflets, (**d**) skirt, and (**e**) final and full geometries of the 23 mm, 26 mm, 29 mm, and 34 mm Medtronic Evolut-R devices.

To generate a realistic TAV geometry, high-resolution micro-CT scanning can be implemented [21,22,24,25,26,29]; however, this technology has limitations such as high cost and accessibility. Even with high-resolution micro-CT, the skirt and prosthetic valves of the TAV device cannot be fully segmented. Therefore, a frequently preferred method was chosen to generate the 3D geometries of the 23 mm, 26 mm, 29 mm, and 34 mm Medtronic Evolut-R TAV parts (stent, skirt, and prosthetic leaflets) as shown in Figure 3, based on the dimensions provided by the manufacturer [16,23,27,30,31,32,33]. The front-view image of each Medtronic Evolut-R device from the catalog was imported into SolidWorks, and a multi-point spline was constructed to generate the profile shown in Figure 3a of the devices from the image, calibrated in height and diameter based on the catalog values. The quantity and size of the stent’s 2D diamond shape patterns were designed using catalog images and the size of the obtained device’s stent profile. To achieve the final 3D shape of the stent illustrated in Figure 3b, the stent’s 2D shape was extruded up to 0.3mm and then projected on a 2D surface generated by a revolving spline profile around the centerline. The skirt and prosthetic valves’ (Figure 3c,d) surface geometries were also generated based on catalog values, images, and generated stent geometries, then extruded to 0.2 mm.

### 2.2. Meshing Methodology and Convergency Analysis

**Figure 4:**
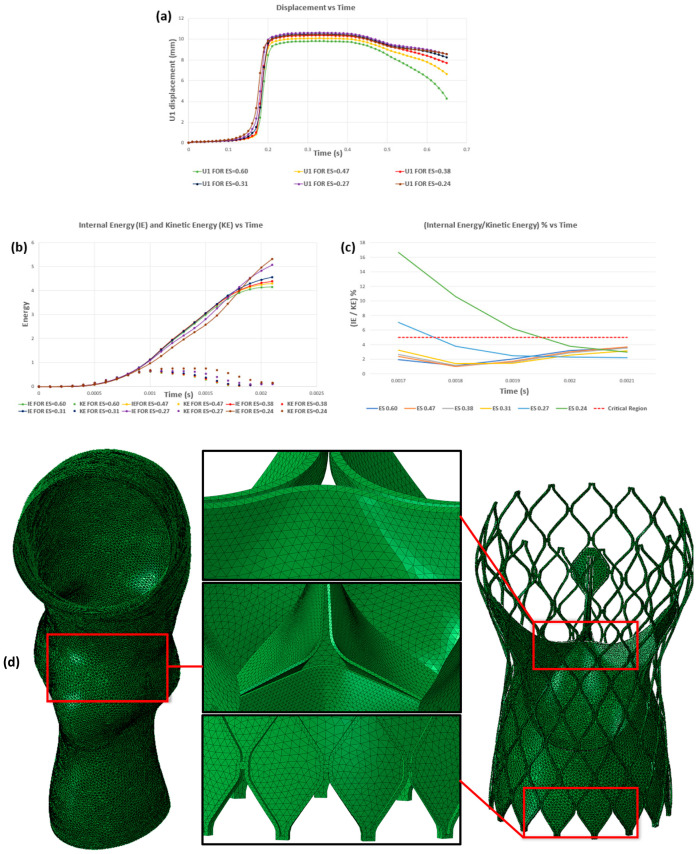
**Mesh convergence analysis graphs for prosthetic leaflets.** (**a**) U1 displacement versus time plot for the upper midpoint of the prosthetic leaflet at different element sizes, (**b**) internal energy (IE) and kinetic energy (KE) versus time plot for the prosthetic leaflet at different element sizes, and (**c**) the percentage of IE over KE for the prosthetic leaflet at different element sizes. (**d**) The illustration of the meshed aorta, native leaflets, stent, skirt, and prosthetic leaflet geometries.

The selection of proper element type and size during meshing of a geometry are both crucial for the computational efficiency of any finite element analysis. Since Abaqus’s (Dassault Systèmes, 2023) explicit solver calculates the stable time step increment based on the smallest element size, using a very fine mesh directly affects the total analysis time and the computational cost of prosthetic leaflets depending on the element size and number of elements, as shown in Table 2. The appropriate element size of the prosthetic leaflet was determined by ensuring that the average displacement error of the same prosthetic valve (shown in Figure 4a) is less than 7%, which is the widely accepted computational analysis error value, and that the internal energy/kinetic energy ratio (shown in Figure 4b,c) is less than 5%, which is considered safe for quasi-static analyses. In the same way, all mesh convergence analyses were performed separately for aortic and native valves, stent, and skirt, and the optimum element size was selected for each part.

Following the mesh convergence analysis, an example of a TAV is presented in Figure 4d. In this instance, 2D first-order tetrahedral elements with a maximum element size of 0.6 mm were employed for the aortic root, 3D first-order tetrahedral elements with a maximum element size of 0.4 mm were utilized for the TAV stent, TAV skirt, and native AV leaflets, and 0.31 mm was identified as the optimal value for the FE mesh of the prosthetic leaflets. The FE meshes for all components were generated using Altair Hypermesh (version 2011; Altair Engineering Inc., Troy, MI, USA). To ensure the quality of all meshed geometries, the Tetra collapse value was maintained at a value above 0.3.

### 2.3. Preparation of the FEA Input

**Figure 5:**
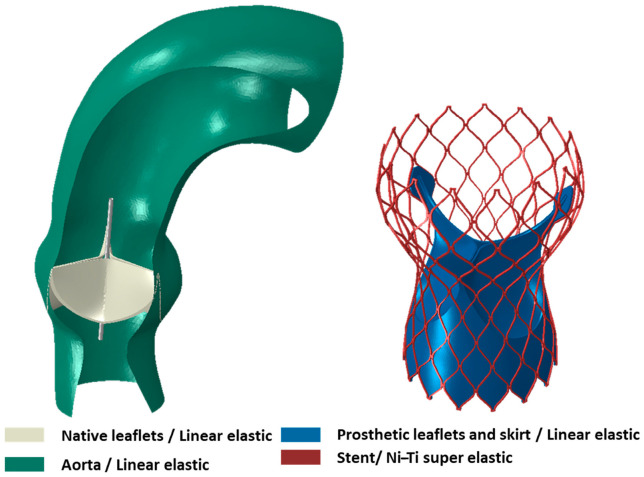
Material behaviors for the aortic root, native leaflets, and TAV device (color-coded).

FE simulations performed in Abaqus (Dassault Systèmes, 2023) include TAV devices (as shown in Figure 5) with stent, skirt, and prosthetic leaflet geometries and patient-specific aorta and native leaflet geometries. The material properties were chosen from literature as super elastic Ni–Ti alloy for the stent, and linear elastic behavior for the aorta [10,21,23,24,30,32,34,35,36], native valve leaflets [21], and prosthetic valve leaflets [23,27,28,29,30,31,32,33], as shown in Table 3. The leaflets of both the native and prosthetic valves, as well as the skirt and stent materials, were defined as homogenous solids. The crimper that crimps the TAV to the desired diameter and the opener, which opens the native valves to a diameter slightly larger than the crimping diameter, were defined as a surface. Additionally, all patient-specific aortic geometries that were imported as surfaces were defined as homogenous shell geometries with a thickness of 2 mm.

### 2.4. Computational Details for the TAV Implantation Procedure and Post-TAVI FEA

The TAV implantation process involves two main structures: the aorta and the TAV. These structures are further subdivided into the aortic root, native valves, stent, skirt, and prosthetic leaflets. To visualize the FEA-calculated parameters for the geometry of each of these structures, the geometries were imported separately. In the entire FEA, ten distinct geometries were under interest, including an aorta, three native leaflets, three prosthetic leaflets, a skirt, a stent, and an opener. All TAV geometries were assembled into patient-specific aortic geometries, with the stent base surface placed 4 mm below the plane of the annulus shown in Figure 2b, after aligning the prosthetic leaflets and native leaflets consecutively.

The clinical TAVI procedure involves crimping the TAV and inserting it into the catheter, guiding the catheter into the heart, and inserting it into the opening of the aortic valve. These procedures were converted to three distinct stages in FEA: native valve opening, crimping, and deployment. Since these steps are associated with large deformation even though they are not under the influence of dynamic forces, quasi-static simulations were performed by using Abaqus’s (Dassault Systèmes, 2023) explicit solver. During the opening step, a cylindrical surface geometry was used to expand the native valves in the radial direction up to the crimping diameter. This step lasted for 0.2 s and had a mass scaling with a target time increment value of 4 × 10^−6^. In the crimping step, the TAV was crimped with a cylindrical crimper which had a slightly larger diameter than the TAV itself, to a size suitable for deployment into the native valve. During the deployment step, the crimped TAV was released from the crimper and was inserted into the native aortic valve. Those crimping and deployment steps lasted for 1.2 s in total and had a mass scaling with a target time increment value of 2 × 10^−6^ with a frequency of every 2000 increments. Since all geometries were independent of each other, a tie constraint was enforced to define geometries that were in contact with each other. A surface-to-surface tie constraint was used to connect the aorta with native leaflets, skirt with prosthetic leaflets, and skirt with stent. Additionally, frictionless general contact properties were used for all steps.

The motion-limiting boundary conditions defined on the aorta and TAV stent were included in all steps, and were defined by specifying the start and exit cross-section of the aorta in the global coordinate system as “ENCASTRE” and the base surface of the TAV stent as “ZSYMM”. A positive direction “U1 or UR” was defined on the opener’s surface stent in the cylindrical coordinate system, which had a cylindrical surface geometry with a diameter of 1 mm. This direction was activated in the opening step and deactivated in the deployment step. The opener opened the native valves that were already closed to a slightly larger diameter than the crimping diameter. In conjunction with this, a negative direction “U1 or UR” (which was established on the surface of the crimper) crimped the TAV up to a 14 mm stent in the cylindrical coordinate system; this direction was active during the crimping step and deactivated after the deployment step. In contrast, in the deployment step, this boundary condition was applied in a positive direction. The boundary conditions that existed in the opening, crimping, and deployment steps were applied as the “equally spaced” amplitude. To eliminate energy oscillations, viscous pressure was applied to the outer surface of all geometries, except for the opener and the crimper. To make these steps clear, a complete TAVI simulation for an example case is provided in Supplementary Video S1.

Post-processing assessment was performed following the completion of the FEA using ABAQUS. Contour plots were generated for the following parameters for all cases: contact pressure generated on the aortic root by the stent, von Mises stress on the aortic root tissue, and radial displacement for the aortic root tissue. The maximum limit values were set at 0.4 MPa, 0.6 MPa, and 0.3 mm on the legends, respectively, to facilitate the visualization of the results. The average and maximum contact pressure, von Mises stress, and radial displacement values in the annulus region for all cases were calculated from the stress regions obtained by delimiting the regions above 0.1 MPa, 0.17 MPa, and 0.16 mm, respectively. These region value constraints were applied in order to ensure that the average value calculated over the aorta does not approach zero for the zero-stress regions of the aorta that are not subjected to any stress.

## 3. Results

In this study, 14 patients’ specific virtual TAV implantations were simulated using a total of 40 FEA cases. Each patient-specific case was investigated under three FEA conditions: a real implant, and a smaller and bigger Medtronic Evolut-R TAV. Three different virtual TAV implantation analyses were carried out for each patient-specific case, except for the patients who had 34 mm TAV deployment since there is no bigger size available than the 34 mm.

The results of the TAV stent outer surface contact area, TAV contact pressure on the aorta’s inner surface, von Mises stress on the aorta’s inner surface, and aortic radial displacement are presented in the last time step of all FEAs. The results were grouped under actual implanted TAV sizes of 26 mm, 29 mm, or 34 mm. For each case within the group, results for smaller and larger valve implantation scenarios are also presented. In this way, the effect of different sizes of TAVs on the FEA results could be assessed for each studied patient case. Moreover, the FEA results for patients who underwent the same size valve implantation were compared to examine the influence of aortic geometrical variations.

**Figure 6:**
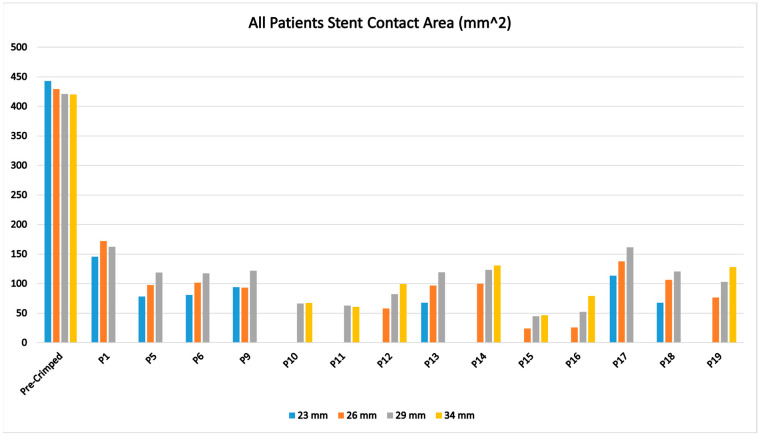
FEA stent contact area results for all examined cases, compared to pre-crimped TAV stent outer areas seen on the left bars.

Contact area measurements of all patients and all different deployment scenarios are compared (in mm^2^) in Figure 6. The contact area was defined as the intersection of the aorta inner surface and the stent outer surface areas where the contact pressure value exceeded 0.0001 MPa. In Figure 6, for each patient, contact areas are presented with three values: the actual implanted valve case in the middle bar, the smaller valve size scenario on the left, and the bigger valve size scenario on the right, except for P10 and P11. For these cases, a 34 mm valve was implanted; its value is shown on the right bar and the smaller valve scenario is shown on left. Four left bars (pre-crimped) represent the total outer surface areas of the stents for comparison. These areas were calculated for the stent geometries before FEA crimping and represent the maximum possible attachment contact areas post-deployment within aorta geometries. In Figure 6, for the majority of the cases, the contact area increases with increased valve size, as expected. However, in some cases, increased valve size did not change contact area (P9, P10, P15) and in some other cases, increased valve size decreased contact area (P1 and P11). These findings provide evidence for the importance of geometrical variations on the attachment of TAVs.

**Figure 7:**
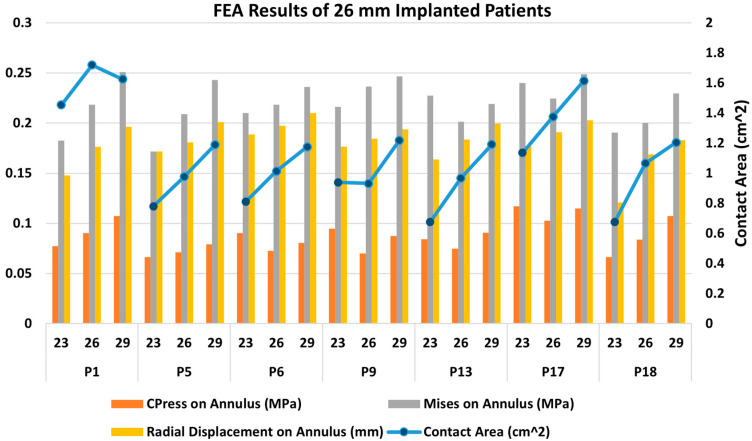
Bar chart representation of the FEA results for 26 mm TAV implanted cases; 23 mm TAV and 29 mm TAV implantation were also simulated for comparison.

**Figure 8:**
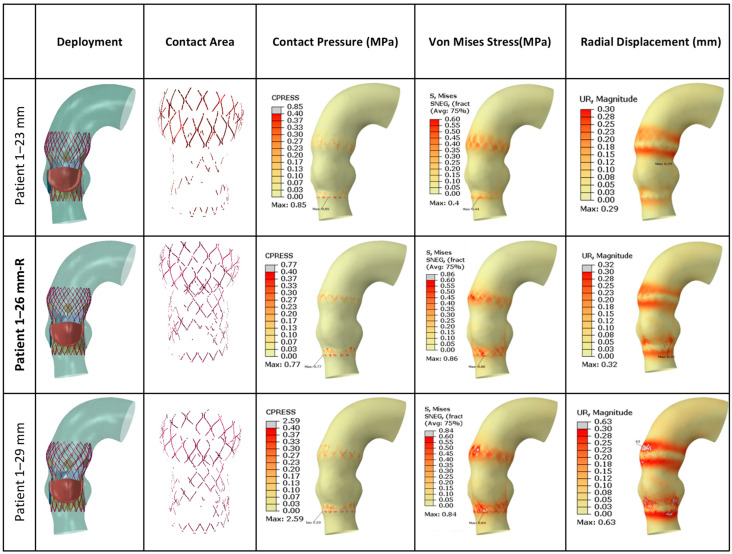
**Graphical presentation of FEA results for 26 mm TAV implanted cases.** The 23 mm TAV and 29 mm TAV implantation were also simulated for comparison for each case. Actual valve sizes for the implants are indicated in bold and designated with “-R”. Contour plots for all 26 mm cases are provided in Supplementary Materials.

Figure 7 presents FEA wall stress and deformation results for the cases where a 26 mm TAV was implanted. For each case, a smaller valve scenario results on the left bar, and a higher valve scenario results on the right bar are also presented. The average annulus contact pressure in MPa, average annulus von Mises stress in MPa, and average annulus radial displacement in mm are presented in bar charts, whereas contact area in cm^2^ is also presented in the figure, as a line graph for reference. All of the seven cases that were studied here have similar annular dimensions, hence the 26 mm TAV was selected to be implanted. Interestingly, there was a large variation in wall stresses and deformations among these cases. The 26 mm actual TAVI FEA simulations revealed that the range of average contact pressure was between 0.070 and 0.102 MPa, the range of average von Mises stress was between 0.200 and 0.237 MPa, the range of average radial displacement was between 0.169 and 0.197 mm, and the range of contact area was between 0.932 and 0.102 cm^2^. When we analyzed TAV size-specific variations for each patient, in most cases von Mises stresses and radial displacement increased with increasing size. For contact area, for all cases except P1 and P9, the contact area increased with valve size. For contact pressure, for three cases (P1, P5, P18), the contact pressure increased with increasing size; for the remaining four cases, no linear relation could be found. It seems as if there is no common trend for any parameter that applies to each studied case. Figure 8 presents post-deployment stent configurations (left column), stent regions that are in contact (second left column), and contour graphs for contact pressure, von Mises stresses, and radial displacements, for the sample case (Patient no. 1), included in Figure 7.

**Figure 9:**
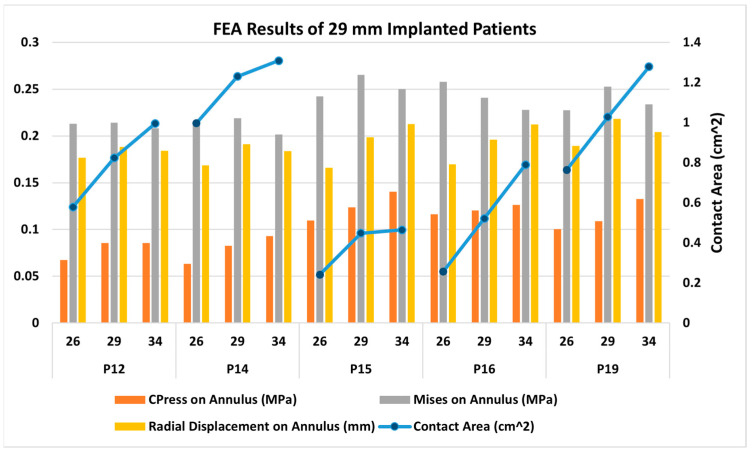
Bar chart representation of the FEA results for 29 mm TAV implanted cases. 26 mm TAV and 34 mm TAV implantation were also simulated for comparison.

**Figure 10:**
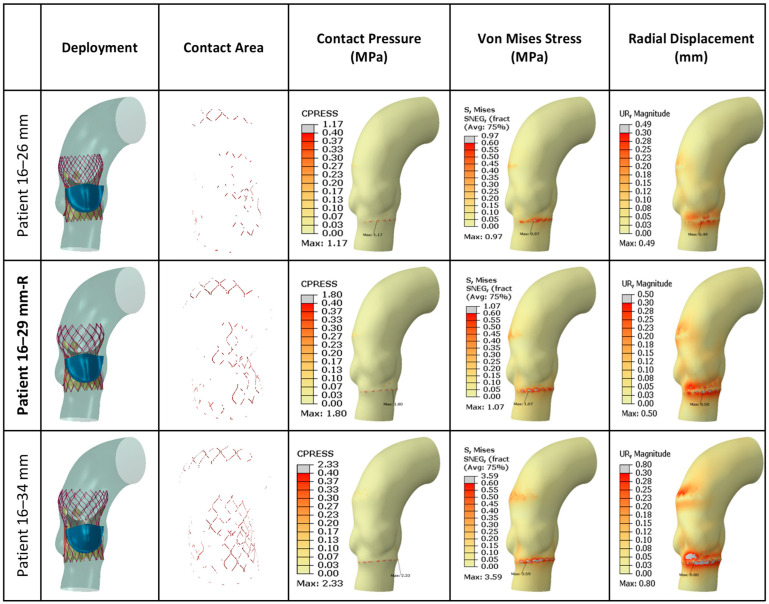
**Graphical representation of FEA results for 29 mm TAV implanted cases**. The 26 mm TAV and 34 mm TAV implantations were also simulated for a comparative analysis in each case. Actual valve sizes for the implants are indicated in bold and designated with “-R”. Contour plots for all 29 mm cases are provided in Supplementary Materials.

Figure 9 presents the FEA wall stress and deformation results for the cases where a 29 mm TAV was implanted. For each case, the smaller valve scenario results are also presented on the left bar, and the higher valve scenario results on the right bar. The average annulus contact pressure in MPa, average annulus von Mises stress in MPa, and average annulus radial displacement in mm are presented in bar charts, whereas the contact area in cm^2^ is also presented as a line graph for reference. All of the five cases that were studied here have similar annular dimensions, hence the 29 mm TAV was selected to be implanted. Similar to the 26 mm cases, there was a large variation in wall stresses and deformations among these cases. The 29 mm actual TAVI FEA simulations revealed that the range of average contact pressure was between 0.082 and 0.124 MPa, the range of average von Mises stress was between 0.214 and 0.265 MPa, the range of average radial displacement was between 0.188 and 0.218 mm, and the range of contact area was between 0.448 and 1.230 cm^2^. When we analyzed the TAV size-specific variations for each patient, in all cases, the stent contact area as well as the contact pressure increased with valve size. The von Mises stresses and radial displacement does not seem to be correlated with valve size. Figure 10 presents the post-deployment stent configurations (left column), stent regions that are in contact (second left column), and contour graphs for contact pressure, von Mises stresses, and radial displacements, for the sample case (Patient no. 16), included in Figure 9.

**Figure 11:**
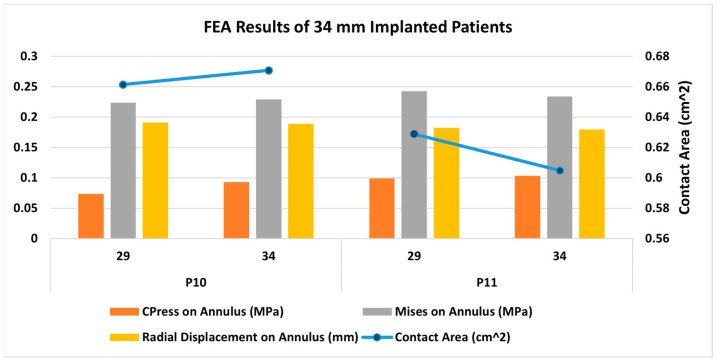
Bar chart representation of the FEA results for 34 mm TAV implanted cases. The 29 mm TAV implantation was also simulated for comparison.

**Figure 12:**
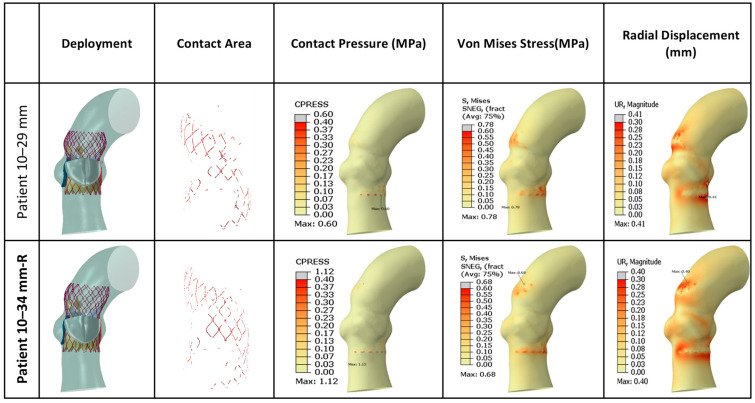
Graphical representation of FEA results for 34 mm TAV implanted cases. The 29 mm TAV implantation was also simulated for a comparative analysis in each case. Actual valve sizes for the implants are indicated in bold and designated with “-R”. Contour plots for all 34 mm cases are provided in Supplementary Materials.

Figure 11 presents FEA wall stress and deformation results for the cases where a 34 mm TAV was implanted. For each case, a smaller valve scenario result on the left bar is also presented. The average annulus contact pressure in MPa, average annulus von Mises stress in MPa, and average annulus radial displacement in mm are presented in bar charts, whereas the contact area in cm^2^ is also presented as a line graph for reference. Both of the cases that were studied here have similar annular dimensions, hence the 34 mm TAV was selected to be implanted. Since there are few cases here, there were fewer variations in wall stresses and deformations among these cases. The 34 mm actual TAVI FEA simulations revealed that the range of average contact pressure was between 0.093 and 0.103 MPa, the range of average von Mises stress was between 0.229 and 0.234 MPa, the range of average radial displacement was between 0.179 and 0.189 mm, and range of contact area was between 0.604 and 0.670 cm^2^. When we analyzed the TAV size-specific variations for each patient, in both cases, the stent contact area increased with valve size. Contact pressure, von Mises stresses, and radial displacement do not seem to change much with the valve size. Figure 12 presents post-deployment stent configurations (left column), stent regions that are in contact (second left column), and contour graphs for contact pressure, von Mises stresses, and radial displacements, for the sample case (Patient no. 10), included in Figure 11.

## 4. Discussion

TAVI has revolutionized AV therapy in the last 20 years. Here, a catheter-guided crimped stented valve was guided into the degenerative native valve and deployed to relieve severe aortic stenosis. As patient populations for TAVI expand, varieties in patient anatomies due to different ages will become evident. Moreover, aortic disease severities will also vary significantly from patient to patient. For example, the degree of valve calcification, presence of bicuspid anatomy, etc., are known to closely influence TAVI outcomes [37]. Despite these wide variabilities in diseased aortic valve conditions, at the clinical practice, there are very few TAV brands, with limited sizes, applied to all patients. Computational modeling is a powerful approach for virtually simulating TAVI within a patient AV geometry for assessing TAV performance. Here, FEA can be used to assess the attachment of the TAV to the aortic root and visualize the resulting stresses and deformations. These are critical in predicting abnormalities post-TAVI, such as conduction problems, valve migration, or paravalvular leakage [38,39]. Pioneering works in this field have investigated utilizing different numerical techniques and different computational tools to simulate TAVI [12]. In most of these works, only a few cases could be examined, mainly as a proof of concept due to difficulties in generating the models and the high cost of computations [40,41]. FEA has the potential to identify optimal TAV for a specific patient prior to implantation and for such an assessment, the influence of aortic anatomy as well as the effect of TAV size on associated wall stresses should be examined. Therefore, this field can be advanced with comprehensive investigations utilizing FEA for a variety of anatomies with different TAVI scenarios. We conducted this work for this purpose, where we included 14 TAVI cases and simulated actual implanted valves as well as different sized valves to reveal the influence of aortic anatomy and the TAV size on wall stresses. We provided detailed information about pre-processing and post-processing steps of our models to support researchers for similar investigations.

The most common complications relevant to TAVI include paravalvular leakage, conduction abnormalities, valve migration, and bleeding [42,43,44]. Since the TAV is anchored to the aortic root by just a pressure force from the stent, the amount of pressure from the stent is extremely critical for valve attachment. However, excessive pressure might lead to bleeding and conduction abnormalities [42,45]. On the other hand, insufficient valve attachment with low pressure might cause paravalvular leakage as well as valve migration [36,44]. Calculating the contact area between the stent’s outer surface and the aortic root might be useful to assess valve attachment. Calculating the contact pressure and von Mises stresses might be useful for assessing wall stresses, whereas the effect of a deployed valve on aortic root deformation can be assessed by calculating aortic wall displacement such as radial displacement. Even though there is no clinical consensus or set of standards regarding the optimal levels, FEA can be utilized for calculating these mechanical stress parameters to assess TAVI success for optimal valve selection and risk prediction in a patient-specific manner. The FEA in this study included seven cases with 26 mm Medtronic Evolut TAVI, five cases with 29 mm Medtronic Evolut TAVI, and two cases with 34 mm Medtronic Evolut TAVI. Table 4 summarizes FEA analysis results for these cases. Graphical representations of these results are presented in previous tables and figures for each valve size. In clinical practice, the selection of the TAV to be implanted is based on several criteria including patient anatomic dimensions such as aortic annular size. Hence, the patients included in this work who have undergone the same size TAVI have close aortic annular sizes. Our FEA results can be analyzed in different ways. We can first look at the results for the cases under each size that was studied (i.e., 26 mm, 29 mm, or 34 mm). Ranges of studied parameters for each group are as follows: the average contact area for the 26 mm cases is 0.932–0.102 cm^2^, 29 mm is 0.448–1.230 cm^2^, 34 mm is 0.604–0.670 cm^2^; the average contact pressure for the 26 mm cases is 0.070–0.102 MPa, 29 mm is 0.082–0.124 MPa, 34 mm is 0.093–0.103 MPa; the average von Mises stress for the 26 mm cases is 0.200–0.237 MPa, 29 mm is 0.214–0.265 MPa, 34 mm is 0.229–0.234 MPa; and the average radial displacement for the 26 mm cases is 0.169–0.197 mm, 29 mm is 0.188–0.218 mm, 34 mm is 0.179–0.189 mm. These results demonstrate there are large variations in wall stresses and deformations from patient to patient for all studied groups. This is due to local geometrical variations in aortic roots. Our results are consistent with previous studies where FEA was applied for post-TAVI mechanical stress examination for different patients. For example, Rocatello et al. examined post-TAVI contact pressures for a group of 12 patients, and their results demonstrated that the maximum contact pressure varied from 0.35 to 0.9 MPa among the patients [17]. Pasta et al. examined post-TAVI von Mises stresses for a group of nine patients, and their results demonstrated that the maximum von Mises stresses varied from 0.06 to 0.85 MPa among the patients [25]. Levels of all studied parameters in our investigation are also consistent with previous studies. Overall, the levels of the parameters studied here are as follows: contact area ~1 cm^2^; average contact pressure ~0.1 MPa; maximum contact pressure ~1.5 MPa; average von Mises stress ~0.2 MPa; maximum von Mises stress 1 MPa; average radial displacement ~0.2 mm; and maximum radial displacement is ~0.5 mm. Post-TAVI levels of these parameters from previous studies are as follows: the contact area level from Nappi et al. is 886 mm^2^ [46]; contact pressure level from Nappi et al. is 6.9 MPa [46], from Rocaetllo et al. is 0.9 MPa [17]; von Mises stress level from Pasta et al. is 1.8 MPa [25], from McGee et al. is 1.1 MPa [19], from Bianchi et al. is 2.5 MPa [16], and from Morganti et al. is 3 MPa [22]. Hence, the overall level of the parameters that have been studied in this paper is consistent with previous studies, suggesting the accuracy of our results.

For predicting TAVI outcomes, it is desirable to assess different valve sizes in a patient-specific manner. This is particularly important for patients having aortic dimensions on the border for recommendations for two different sizes. A larger valve would be expected to have better attachment, preventing valvular migration and paravalvular leakage, but may also increase wall stress and deformation. In order to investigate how valve attachment, wall stresses, and wall deformations are affected by TAV size, for all the cases included in the study, we have performed FEA for a smaller and a larger valve in addition to an actual implanted valve. In this analysis, we had different results for each TAV group. For the 26 mm TAV group, the contact area increased with valve size for five out of seven cases. For contact pressure, for three cases the contact pressure increased with increasing size, whereas for the remaining four cases, no linear relation could be seen. It seems there is no common trend for any parameter that applies to each studied case for this group. For the 29 mm TAV group, for all cases, the stent contact area as well as the contact pressure increased with valve size, whereas the von Mises stresses and radial displacement did not seem to be correlated with valve size. For the 34 mm TAV group, in both cases, the stent contact area increased with valve size, whereas the contact pressure, von Mises stresses, and radial displacement did not seem to change much with the valve size. These results demonstrate that there is no straightforward correlation of valve size with wall stresses and provide further evidence for the influence of patient-specific anatomical variations in post-TAVI mechanical stresses. Unfortunately, there are no previous studies that we can compare our results with where different valve types and sizes were simulated for same patient geometry. Hence, our study is a pioneering investigation for examining the effect of TAV size on wall stresses. Few similar previous investigations have examined the effect of several TAVI procedural parameters on post-TAVI wall stresses with different FEA simulations on the same patient geometries. For example, Auricchio et al. and Bianchi et al. studied the effect of implantation depth positioning (distal or proximal) on post-TAVI stresses [16,30], demonstrating implantation depth significantly influence wall stresses. We believe our work will contribute to such investigations to explore the effect of different patient-specific and procedural parameters on post-TAVI aortic wall mechanics via FEA.

## 5. Limitations

Our study had a few limitations, mainly due to the simplification of the FEA, as follows: Parameterized native valve models: Since it is not possible to visualize all patients’ native valves from CT images, parameterized native valves were generated based on common anatomical measurements. Therefore, the patient-specific valve geometry and biomechanical behavior may not be fully captured, which may affect the accuracy of simulation results. Uniform wall thickness assumption: Uniform wall thickness was used for all geometries, which does not reflect the natural variation in wall thickness in different regions of the aorta and leaflets. This simplification may affect the stress distribution and mechanical response predicted by the model. Material property simplification: Linear elastic material properties were used for all geometries except for the TAVI device stent. This assumption may not accurately represent the complex nonlinear material behavior of biological tissue, which may affect the accuracy of stress and strain predictions. Standardized deployment orientation: The same deployment orientation was used for all cases, which does not account for potential variations in valve positioning during actual procedures. This uniform approach might limit the generalizability of the findings to real-world TAVI scenarios where orientation can vary significantly. Exclusion of coronary arteries and valve interaction: The models excluded detailed representations of the coronary arteries and potential interactions with the implanted valve. This omission may overlook critical aspects of coronary obstruction risks and local hemodynamic effects post-implantation. Ignoring calcification: The FEA models did not account for calcification in either the aorta or native valve leaflets. The presence of calcification can significantly affect the mechanical properties and behavior of the tissues, influencing the interaction between the TAVI device and the surrounding anatomy. This omission may result in discrepancies between the simulated and actual mechanical performance of the implanted valves.

These limitations underscore the need for more advanced modeling techniques and comprehensive data integration to improve the accuracy and applicability of FEA in evaluating TAVI procedures. Addressing these constraints in future research could enhance the predictive reliability of post-implantation performance assessments.

For the most accurate results, patient-specific factors including the above need to be incorporated in the computational models. However, incorporating all of these parameters is extremely challenging, particularly for studies examining multiple cases such as ours where we modeled 40 cases. Hence, we had to apply simplified assumptions to all cases. One of our main focuses in this study was to examine how aortic wall anatomy influences post-TAVI wall stresses, by comparing cases where same size TAVs were implanted. Since in all of the cases the same simplified assumptions were applied, we believe our results could be used to specifically compare these cases in highlighting the influence of aortic wall anatomies. Our other main focus was to see how wall stresses changed in the same aortic wall geometry when different TAVs were implanted. Again, for such cases, since all simulations were based on same simplified assumption, we believe that these results could be used to highlight the influence of TAV size on wall stresses. Overall, while we acknowledge that simplified assumptions might impact the accuracy of our FEA results, as we applied the same assumptions to all models, we believe our results could be used to examine the influence of aortic wall anatomies as well as TAV sizes on post-TAVI stresses.

## 6. Conclusions and Future Studies

In conclusion, FEA is a powerful approach that can be adapted to the assessment of TAVI for predicting postoperative complications and optimal valve selection for specific patients. Anatomical variations in aortic geometries are very important considerations in proper valve selection in terms of valve attachment and mechanical stresses. Enhancement of computational power and the introduction of new solvers would be expected to develop more practical and faster FEA approaches. This would contribute to the efforts to make FEA a clinical tool for TAVI assessment in the near future. Implementing ML approaches is underway to expedite accurate FEA in this area. In this study, we aimed to detail an FEA approach for virtually simulating TAVI in a patient-specific manner and applied our approach to a total of 40 cases. Each step in our approach was explained in detail for interested researchers to effectively and practically adapt our approach. We believe such investigations will support a better understanding of TAV–native aortic wall interactions, the application of computational methods for TAVI planning, as well as designing more advanced TAVs in the future. While at the moment FEA has limited applicability under clinical settings, with the advancements in FEA solvers as well as the adaptation of artificial intelligence tools to FEA, such computational simulations would be expected to be adapted to clinical settings, including TAVI, for clinical decision making.

To complement the current investigation, in the future we plan to computationally simulate different TAVs such as the balloon-expandable Edwards Sapien in addition to the self-expandable Medtronic Evolut, for a better understanding of the mechanical interactions between implanted TAVs and the aortic wall, incorporating the influence of different types of TAVs. Also, another important aspect is experimentally validating TAVI FEA simulations. For this purpose, phantom geometries can be produced via techniques such as 3D printing. Stress and deformation sensors in the system can be used to measure deformation and wall stresses on aortic wall phantom geometries following the deployment of a TAVI stent within the geometry, which is planned as a future study.

## Figures and Tables

**Table 1:** Characteristics of the subjects in the study cohort.

Patient	Age	Sex	BMI	Degree of Calcification	TAV Size (mm)
P1	60	M	26.0	moderate	26
P5	87	M	27.0	severe	26
P6	87	M	23.7	severe	26
P9	70	F	27.3	severe	26
P10	92	M	NA	severe	34
P11	75	M	34.6	severe	34
P12	76	M	31.7	severe	29
P13	67	F	30.1	severe	26
P14	75	M	27.6	severe	29
P15	93	M	24.8	severe	29
P16	69	F	51.4	severe	29
P17	85	M	33.2	severe	26
P18	81	F	40.7	severe	26
P19	61	M	NA	severe	29

**Table 2:** The dependence of the computational cost of prosthetic leaflets on the element size and number of elements.

Element Size	Number of Elements	Total Analysis Time (s)	Time Increase Factor Compared to 0.6
0.60	4090	102	-
0.47	6360	351	3.44
0.38	10,041	470	4.61
0.31	15,414	667	6.53
0.27	23,383	851	8.34
0.24	35,120	1139	11.16

**Table 3:** Material properties of the aorta, native leaflets, stent, skirt, and prosthetic leaflets.

Stent-Nitinol		Aorta	
Density (tonne·mm^−3^)	6.45 × 10^−9^	Density (tonne·mm^−3^)	2 × 10^−9^
Austenite Young’s modulus (MPa)	51,700	Young’s modulus (MPa)	2
Austenite Poisson’s Ratio	0.3	Poisson’s ratio	0.45
Martensite Young’s modulus (MPa)	47,800		
Martensite Poisson’s Ratio	0.3		
Transformation strain	0.063	**Native valve leaflets**	
Start of transformation (Loading) (MPa)	600	Density (tonne.mm^−3^)	1.1
End of transformation (Loading) (MPa)	670	Young’s modulus (MPa)	8
Start of transformation (Unloading) (MPa)	288	Poisson’s ratio	0.45
End of transformation (Unloading) (MPa)	254		
Start of transformation in compression (Loading) (MPa)	900		
Reference temperature (K)	310	**Prosthetic leaflets and skirt**	
Loading	6.527	Density (tonne.mm^−3^)	1.1
Unloading	6.527	Young’s modulus (MPa)	1
Volumetric transformation strain	0.063	Poisson’s ratio	0.45

**Table 4:** The summary of FEA results: contact area, contact pressure, von Mises stress, and radial displacement for implanted TAVI devices across the inner surface of all patients’ aorta.

Patient	TAV Sizes (mm)	C.A. * (cm^2^)	AVG CPRESS * (MPa)	MAX CPRESS * (MPa)	AVG Mises * (MPa)	MAX Mises * (MPa)	AVG R.D. * (mm)	MAX R.D. * (mm)
P1	26	1.72	0.08	0.77	0.22	0.86	0.18	0.32
P5	26	0.98	0.07	0.54	0.21	0.49	0.18	0.46
P6	26	1.01	0.07	0.81	0.24	0.81	0.20	0.50
P9	26	0.93	0.07	1.16	0.22	1.09	0.18	0.46
P10	34	0.67	0.07	1.12	0.22	0.68	0.18	0.40
P11	34	0.6	0.08	1.32	0.22	0.78	0.18	0.37
P12	29	0.82	0.07	1.04	0.22	0.70	0.19	0.37
P13	26	0.97	0.07	0.80	0.21	0.45	0.18	0.47
P14	29	1.23	0.07	1.03	0.23	0.49	0.19	0.36
P15	29	0.45	0.09	1.47	0.26	1.13	0.20	0.51
P16	29	0.52	0.09	1.80	0.22	1.07	0.18	0.50
P17	26	1.38	0.08	1.18	0.22	1.04	0.18	0.42
P18	26	1.07	0.07	0.87	0.19	0.77	0.16	0.40
P19	29	1.03	0.09	1.49	0.23	1.46	0.20	0.53

* C.A.: contact area; * CPRESS: contact pressure; * Mises: von Mises stress; * R.D.: radial displacement.

## Data Availability

The original contributions presented in this study are included in the article/supplementary material. Further inquiries can be directed to the corresponding authors.

## Supplementary Materials

The following supporting information can be downloaded at: www.mdpi.com/article/10.3390/jcm14030850/s1, Table S1: Graphical presentation of FEA results for 26 mm TAV implanted cases; Table S2: Graphical representation of FEA results for 29-mm TAV implanted cases; Table S3: Graphical representation of FEA results for 34-mm TAV implanted cases; Video S1: Supplementary Video—A complete TAVI simulation.
