# Supplementary material for: Finite Element Analysis of Evolut Transcatheter Heart Valves: Effects of Aortic Geometries and Valve Sizes on Post-TAVI Wall Stresses and Deformations
Source: J Clin Med. 2025 Jan 27;14(3):850. doi: 10.3390/jcm14030850 (PMC11818669; doi:10.3390/jcm14030850)
Supplement: Supplementary file 1 [file jcm-14-00850-s001.zip › Table S1-S3.pdf]

Table S1. Graphical presentation of FEA results for 26 mm TAV implanted cases. 23 mm TAV and 29 mm TAV implantation were also simulated for comparison for each case. Actual valve sizes for the implants are indicated in bold and designated with “-R”.

<table>
<tr><th></th><th>DEPLOYMENT</th><th>CONTACT AREA</th><th>CONTACT PRESSURE (MPa)</th><th>VON MISES STRESS (MPa)</th><th>RADIAL DISPLACEMENT (mm)</th></tr>
<tr><td>Patient 1-23mm</td><td>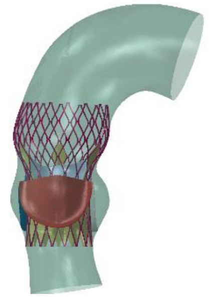</td><td>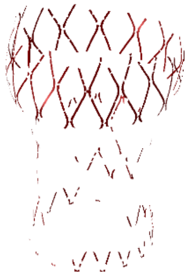</td><td>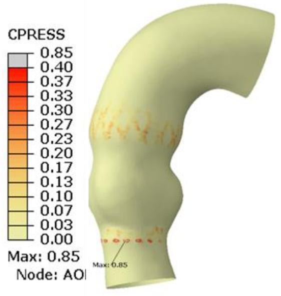</td><td>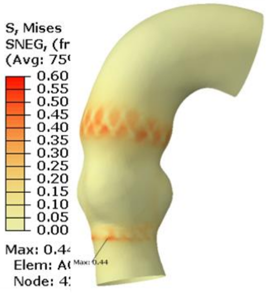</td><td>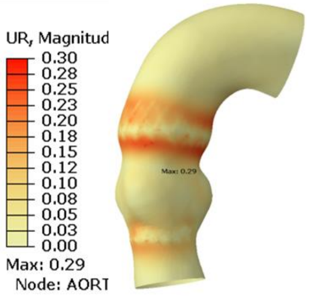</td></tr>
<tr><td>**Patient 1-26mm-R**</td><td>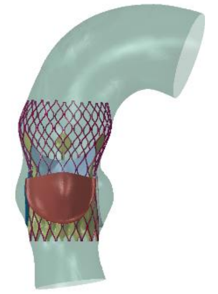</td><td>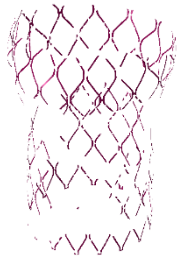</td><td>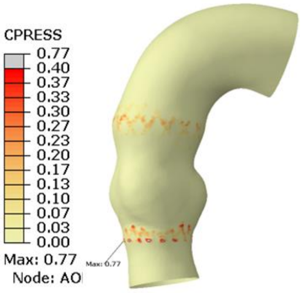</td><td>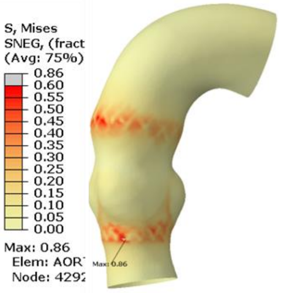</td><td>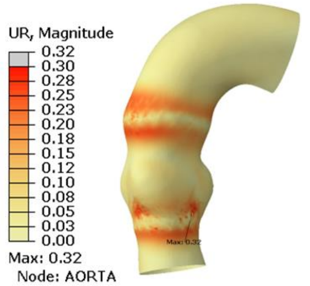</td></tr>
<tr><td>Patient 1-29mm</td><td>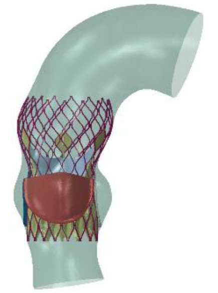</td><td>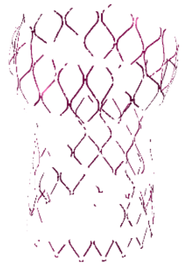</td><td>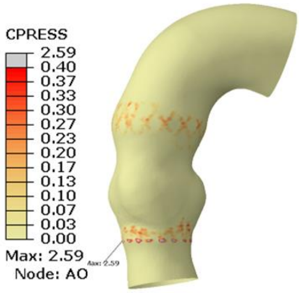</td><td>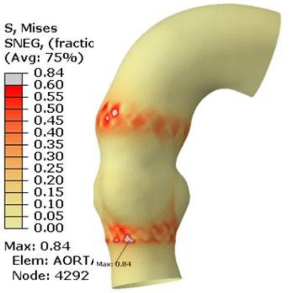</td><td>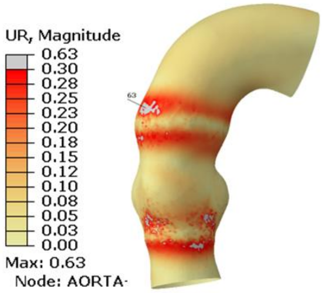</td></tr>
<tr><td>Patient 5-23mm</td><td>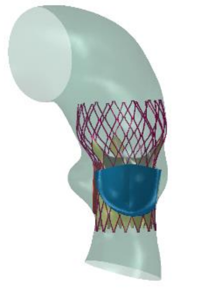</td><td>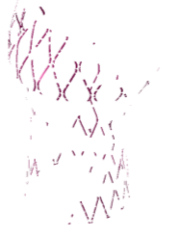</td><td>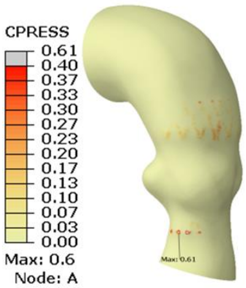</td><td>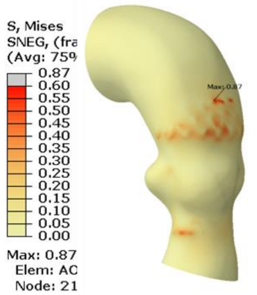</td><td>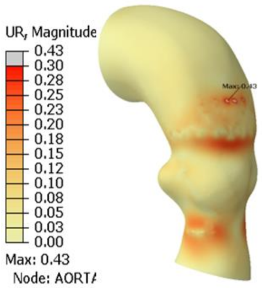</td></tr>
</table>

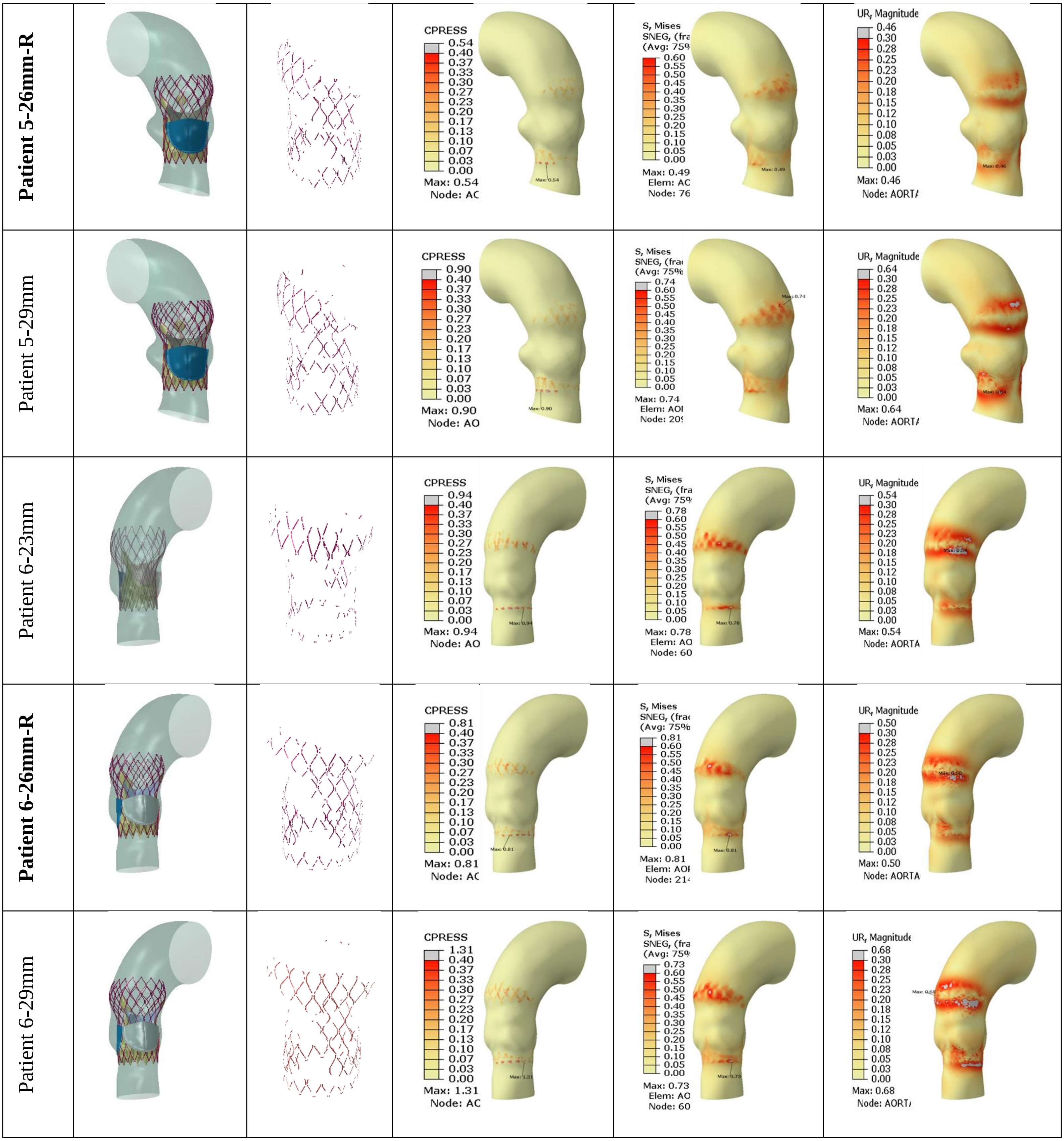

Patient 5-26mm-R
CPRESS
Max: 0.54
Node: AC
S, Mises
SNEG, (fra
(Avg: 75%
Max: 0.49
Elem: AC
Node: 76
UR, Magnitude
Max: 0.46
Node: AORTA
Patient 5-29mm
CPRESS
Max: 0.90
Node: AO
S, Mises
SNEG, (frac
(Avg: 75%
Max: 0.74
Elem: AOI
Node: 20
UR, Magnitude
Max: 0.64
Node: AORTA
Patient 6-23mm
CPRESS
Max: 0.94
Node: AO
S, Mises
SNEG, (fra
(Avg: 75%
Max: 0.78
Elem: AO
Node: 60
UR, Magnitude
Max: 0.54
Node: AORTA
Patient 6-26mm-R
CPRESS
Max: 0.81
Node: AC
S, Mises
SNEG, (frac
(Avg: 75%
Max: 0.81
Elem: AOI
Node: 21
UR, Magnitude
Max: 0.50
Node: AORTA
Patient 6-29mm
CPRESS
Max: 1.31
Node: AC
S, Mises
SNEG, (fra
(Avg: 75%
Max: 0.73
Elem: AO
Node: 60
UR, Magnitude
Max: 0.68
Node: AORTA

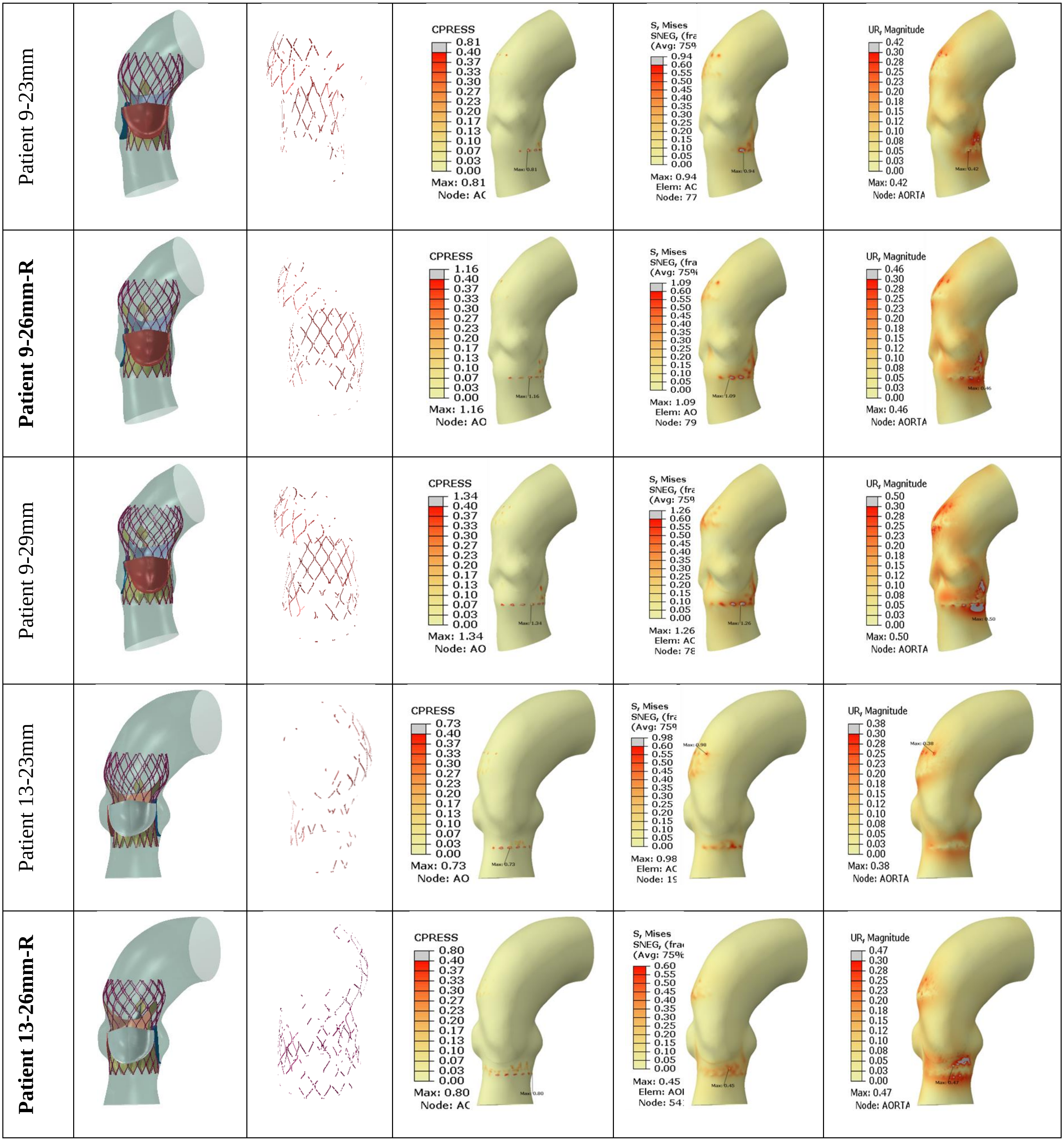

Patient 9-23mm
CPRESS
Max: 0.81
S, Mises
Max: 0.94
UR, Magnitude
Max: 0.42
Node: AORTA
Patient 9-26mm-R
CPRESS
Max: 1.16
S, Mises
Max: 1.09
UR, Magnitude
Max: 0.46
Node: AORTA
Patient 9-29mm
CPRESS
Max: 1.34
S, Mises
Max: 1.26
UR, Magnitude
Max: 0.50
Node: AORTA
Patient 13-23mm
CPRESS
Max: 0.73
S, Mises
Max: 0.98
UR, Magnitude
Max: 0.38
Node: AORTA
Patient 13-26mm-R
CPRESS
Max: 0.80
S, Mises
Max: 0.45
UR, Magnitude
Max: 0.47
Node: AORTA

Patient 13-29mm

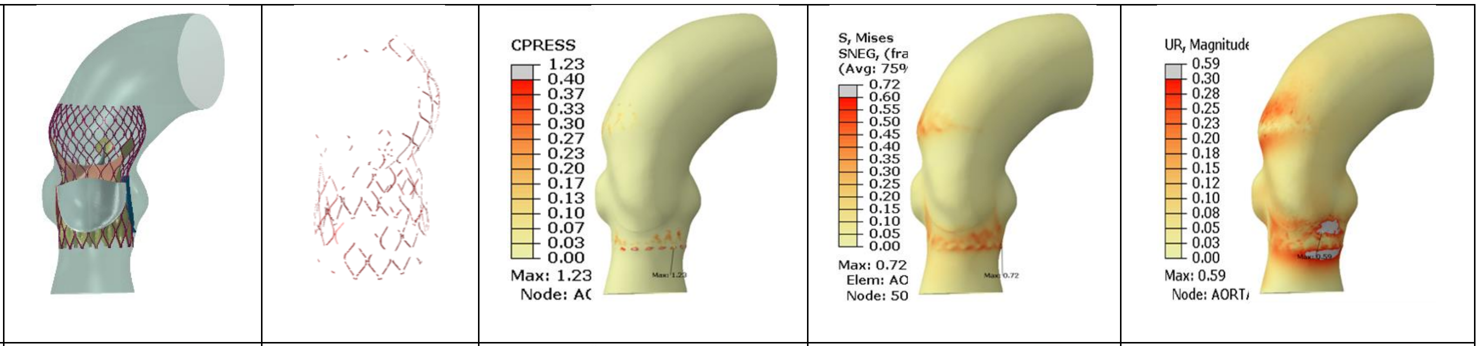

Patient 17-23mm

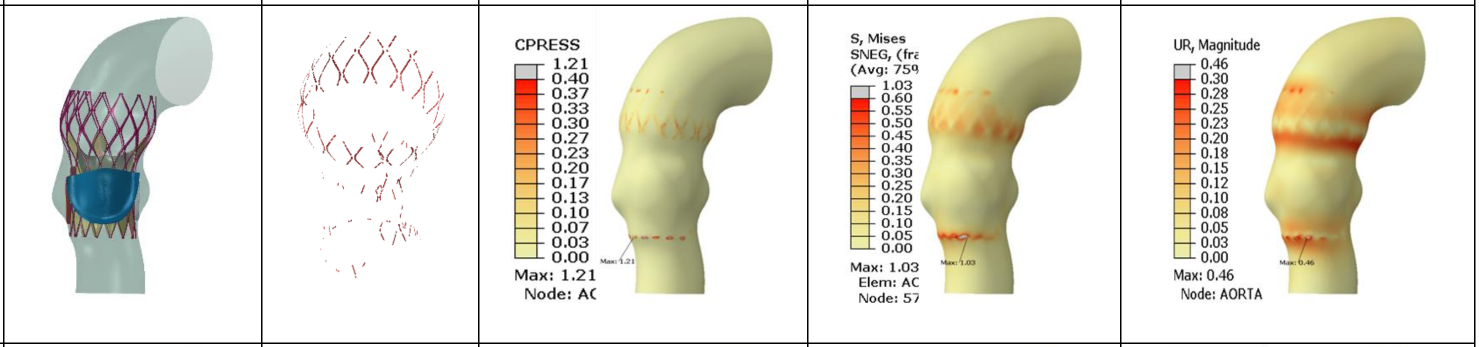

Patient 17-26mm-R

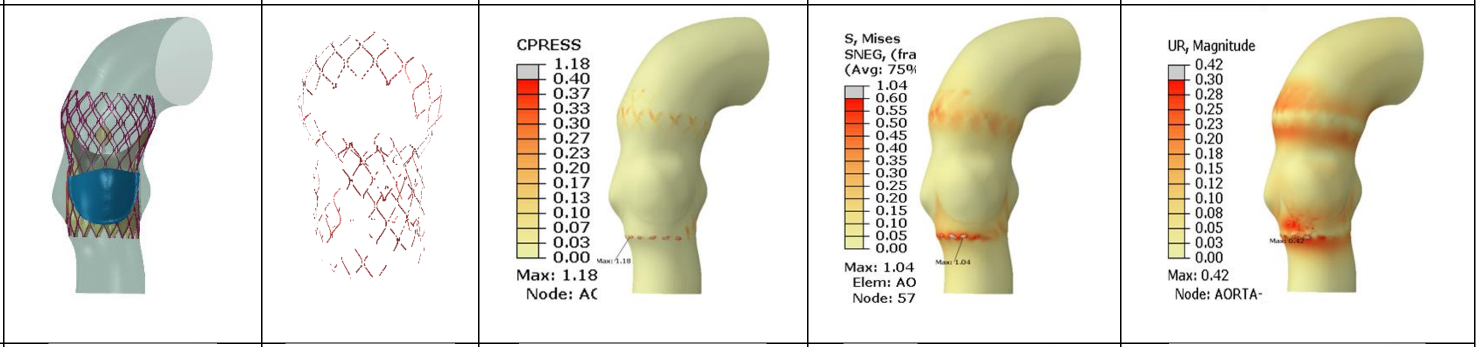

Patient 17-29mm

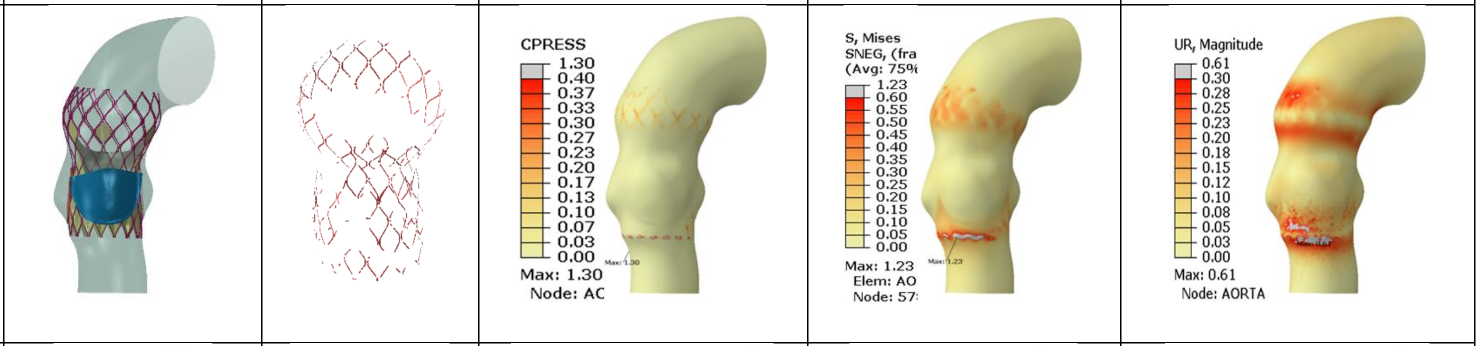

Patient 18-23mm

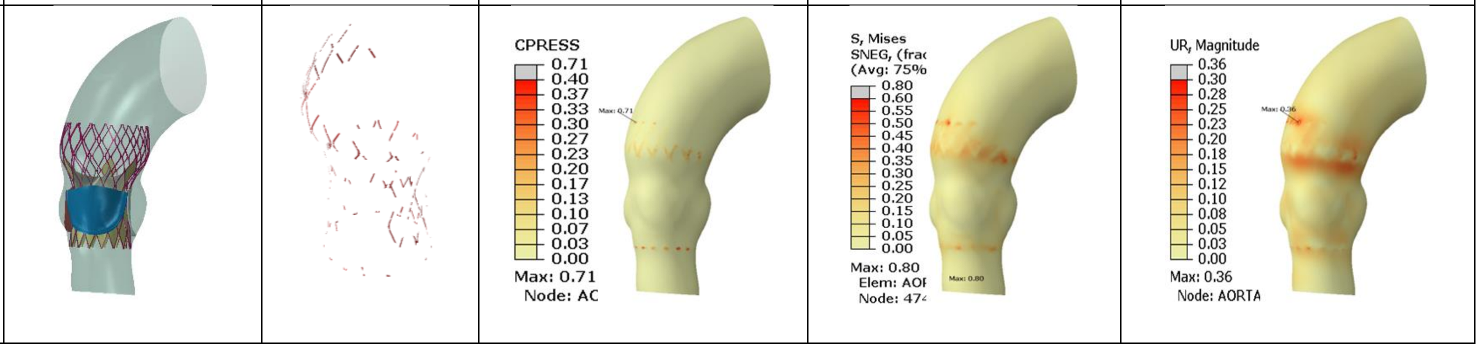

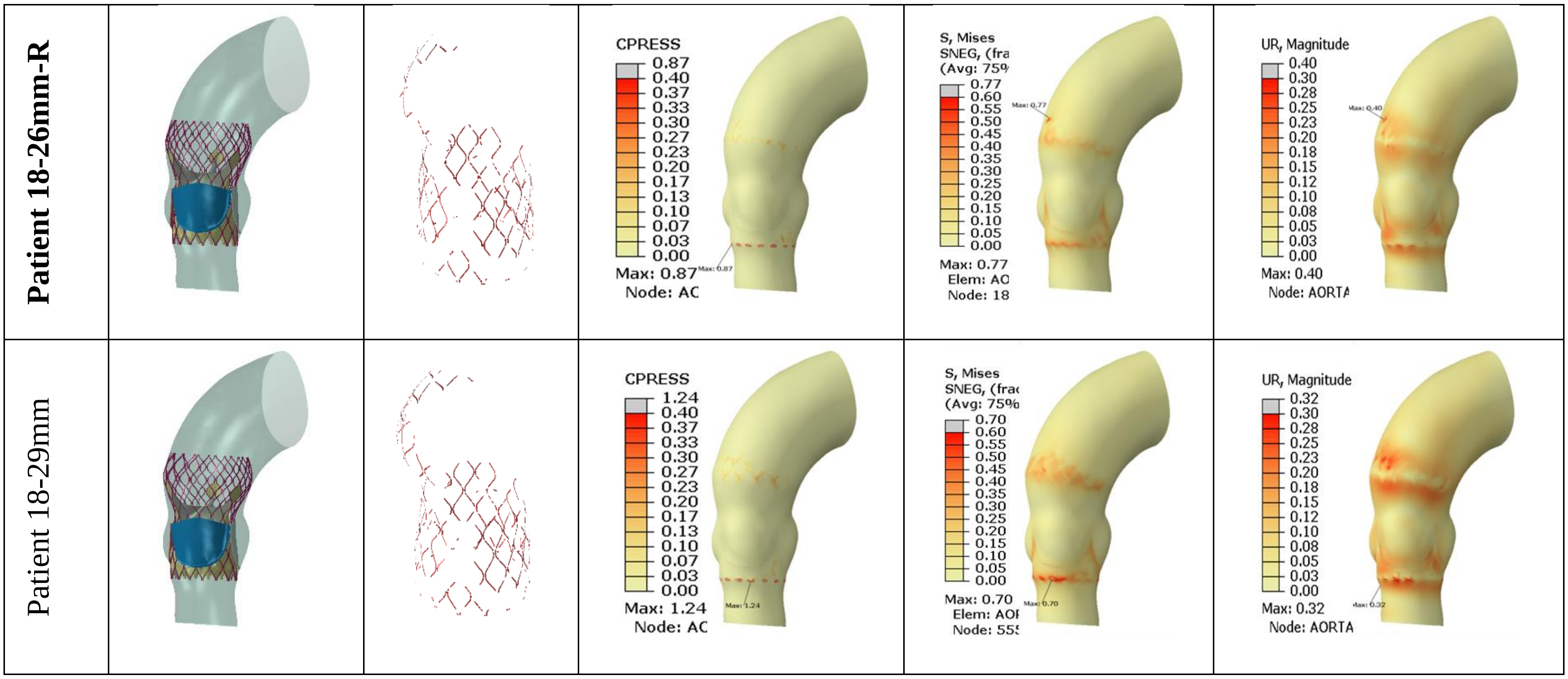
Patient 18-26mm-R
CPRESS
0.87
0.40
0.37
0.33
0.30
0.27
0.23
0.20
0.17
0.13
0.10
0.07
0.03
0.00
Max: 0.87
Node: AC
S, Mises
SNEG, (fra
(Avg: 75
0.77
0.60
0.55
0.50
0.45
0.40
0.35
0.30
0.25
0.20
0.15
0.10
0.05
0.00
Max: 0.77
Elem: AO
Node: 18
UR, Magnitude
0.40
0.30
0.28
0.25
0.23
0.20
0.18
0.15
0.12
0.10
0.08
0.05
0.03
0.00
Max: 0.40
Node: AORTA
Patient 18-29mm
CPRESS
1.24
0.40
0.37
0.33
0.30
0.27
0.23
0.20
0.17
0.13
0.10
0.07
0.03
0.00
Max: 1.24
Node: AC
S, Mises
SNEG, (frac
(Avg: 75%
0.70
0.60
0.55
0.50
0.45
0.40
0.35
0.30
0.25
0.20
0.15
0.10
0.05
0.00
Max: 0.70
Elem: AOR
Node: 55
UR, Magnitude
0.32
0.30
0.28
0.25
0.23
0.20
0.18
0.15
0.12
0.10
0.08
0.05
0.03
0.00
Max: 0.32
Node: AORTA

Table S2. Graphical representation of FEA results for 29-mm TAV implanted cases. 26-mm TAV and 34-mm TAV implantation were also simulated for a comparative analysis in each case. Actual valve sizes for the implants are indicated in bold and designated with "-R".

	DEPLOYMENT	CONTACT AREA	CONTACT PRESSURE (MPa)	VON MISES STRESS (MPa)	RADIAL DISPLACEMENT (mm)
Patient 12-26mm	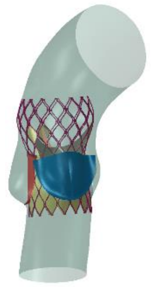	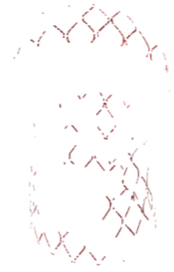	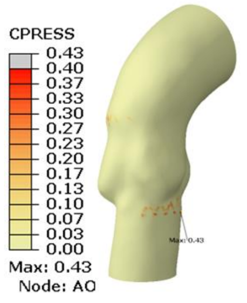	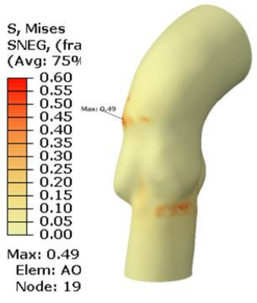	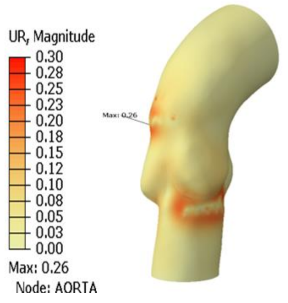
Patient 12-29mm-R	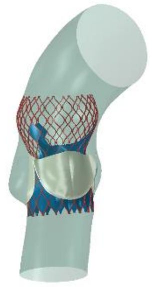	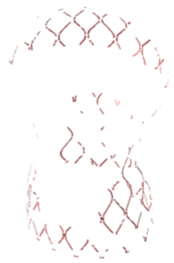	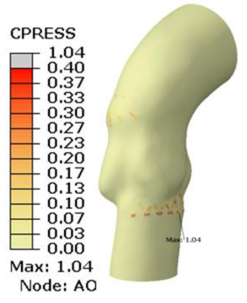	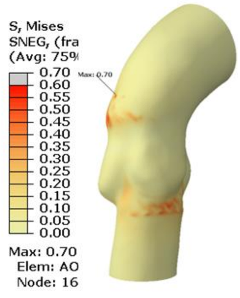	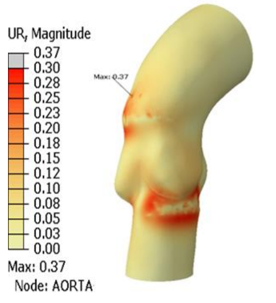
Patient 12-34mm	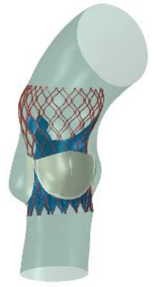	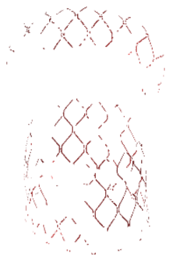	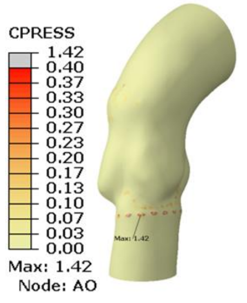	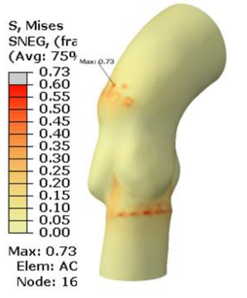	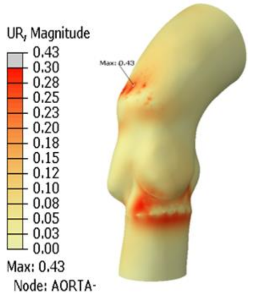
Patient 14-26mm	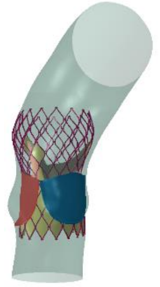	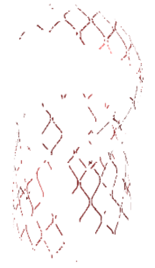	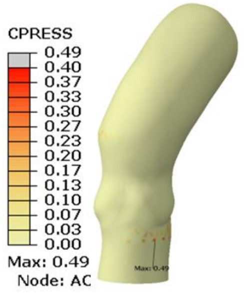	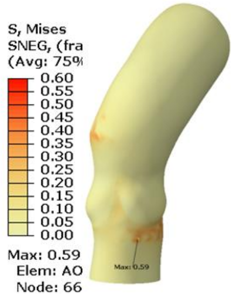	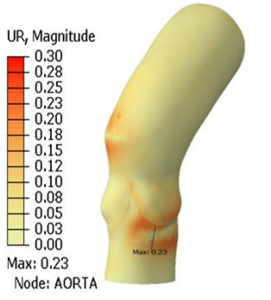

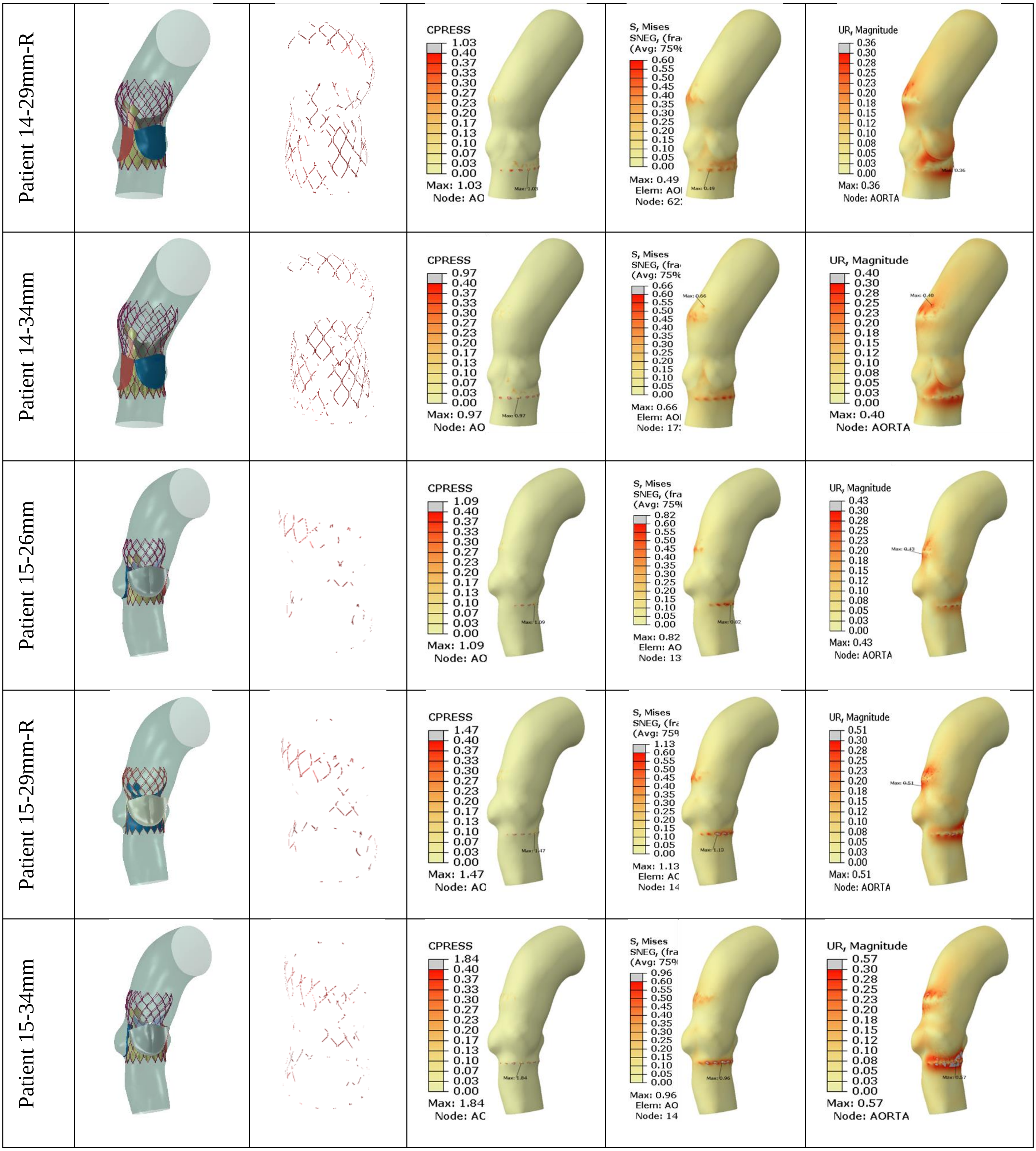

Patient 14-29mm-R
CPRESS
Max: 1.03
Node: AO
S, Mises
SNEG, (fra
(Avg: 75%
Max: 0.49
Elem: AO
Node: 62
UR, Magnitude
Max: 0.36
Node: AORTA
Patient 14-34mm
CPRESS
Max: 0.97
Node: AO
S, Mises
SNEG, (fra
(Avg: 75%
Max: 0.66
Elem: AO
Node: 17
UR, Magnitude
Max: 0.40
Node: AORTA
Patient 15-26mm
CPRESS
Max: 1.09
Node: AO
S, Mises
SNEG, (fra
(Avg: 75%
Max: 0.82
Elem: AO
Node: 13
UR, Magnitude
Max: 0.43
Node: AORTA
Patient 15-29mm-R
CPRESS
Max: 1.47
Node: AO
S, Mises
SNEG, (fra
(Avg: 75%
Max: 1.13
Elem: AC
Node: 14
UR, Magnitude
Max: 0.51
Node: AORTA
Patient 15-34mm
CPRESS
Max: 1.84
Node: AC
S, Mises
SNEG, (fra
(Avg: 75%
Max: 0.96
Elem: AO
Node: 14
UR, Magnitude
Max: 0.57
Node: AORTA

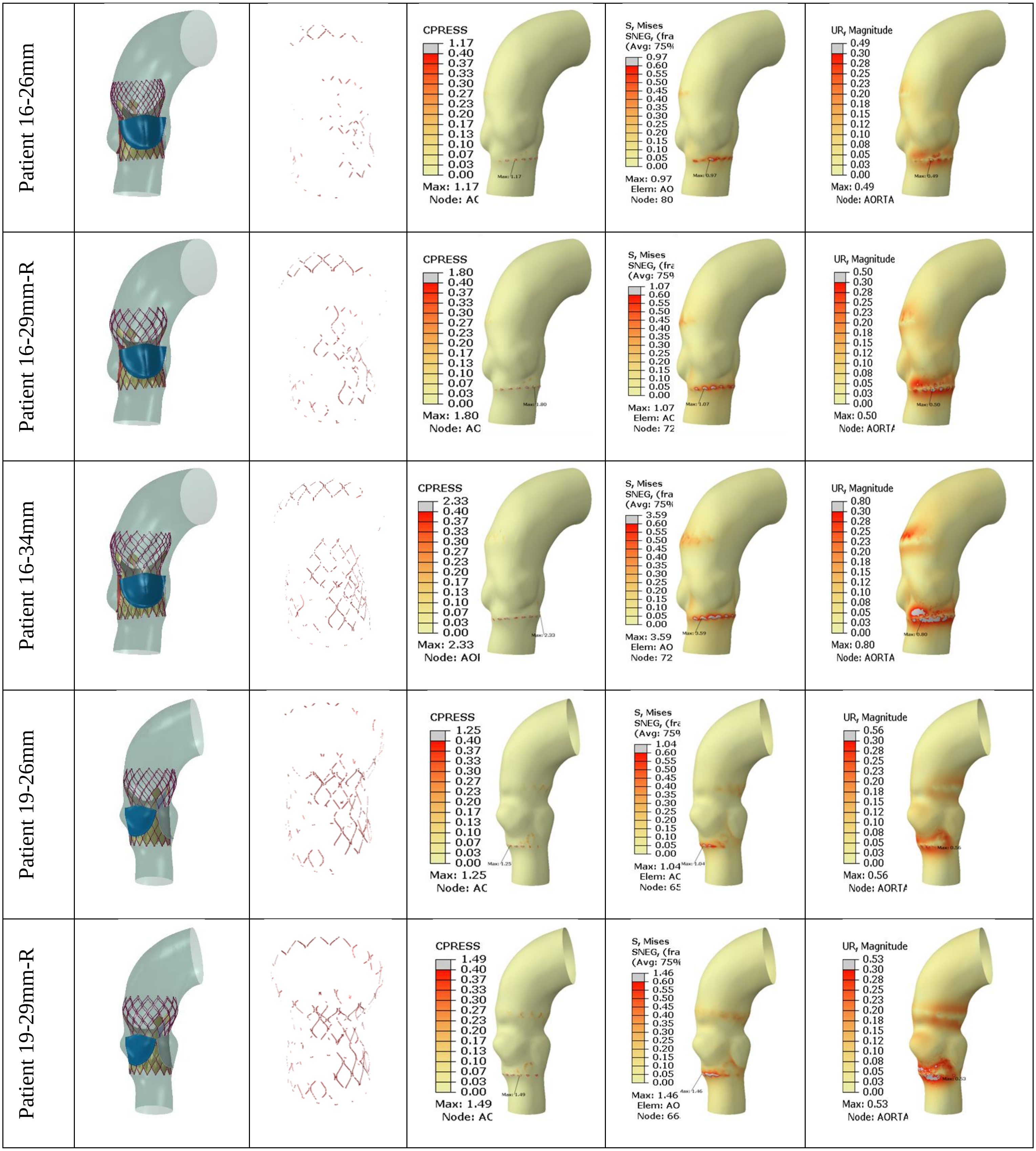
Patient 16-26mm
CPRESS
Max: 1.17
S, Mises
Max: 0.97
UR, Magnitude
Max: 0.49
Patient 16-29mm-R
CPRESS
Max: 1.80
S, Mises
Max: 1.07
UR, Magnitude
Max: 0.50
Patient 16-34mm
CPRESS
Max: 2.33
S, Mises
Max: 3.59
UR, Magnitude
Max: 0.80
Patient 19-26mm
CPRESS
Max: 1.25
S, Mises
Max: 1.04
UR, Magnitude
Max: 0.56
Patient 19-29mm-R
CPRESS
Max: 1.49
S, Mises
Max: 1.46
UR, Magnitude
Max: 0.53

<table>
<tr>
<td>Patient 19-34mm</td>
<td>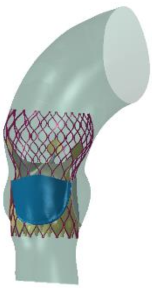</td>
<td>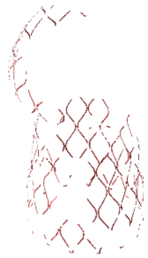</td>
<td>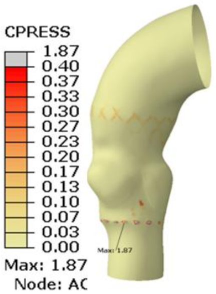
</td>
<td>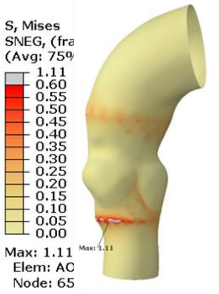
</td>
<td>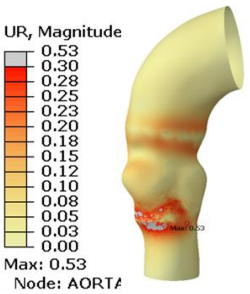
</td>
</tr>
</table>

Table S3. Graphical representation of FEA results for 34-mm TAV implanted cases. 29-mm TAV implantation was also simulated for a comparative analysis in each case. Actual valve sizes for the implants are indicated in bold and designated with "-R".

—	DEPLOYMENT	CONTACT AREA	CONTACT PRESSURE (MPa)	VON MISES STRESS~~C~~(MPa)	RADIAL DISPLACEMENT (mm)
Patient 10-29mm	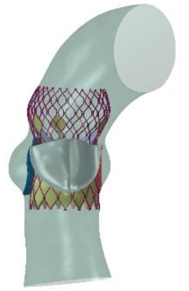	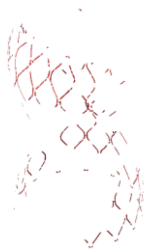	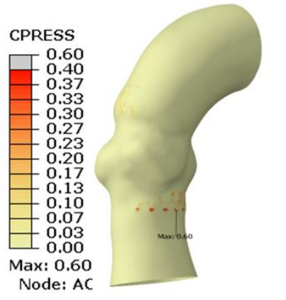	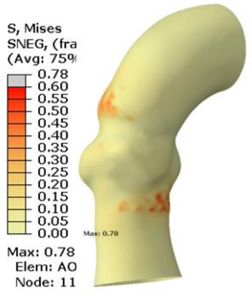	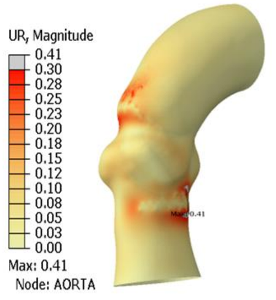
Patient 10-34mm-R	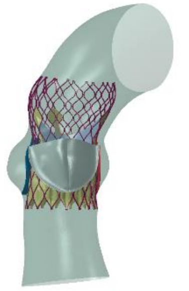	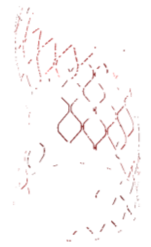	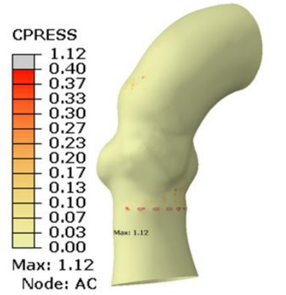	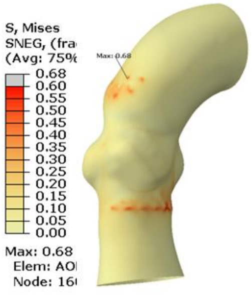	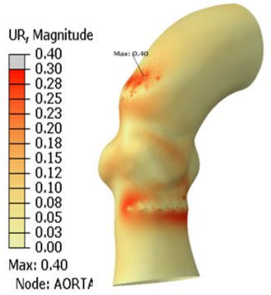
Patient 11-29mm	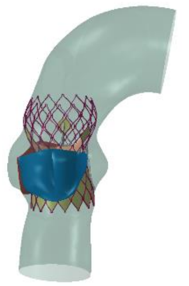	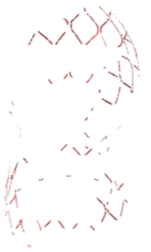	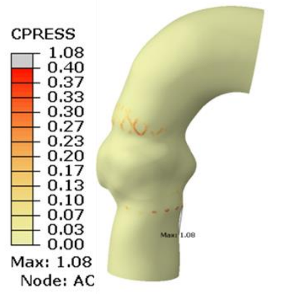	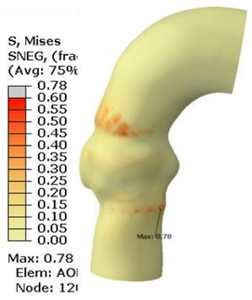	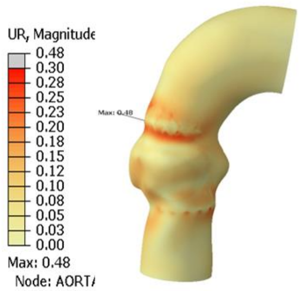
Patient 11-34mm-R	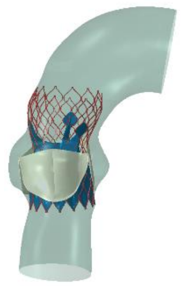	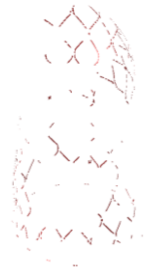	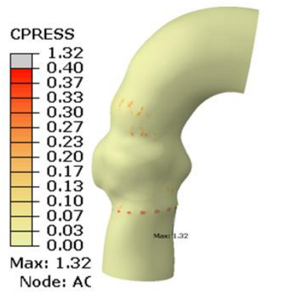	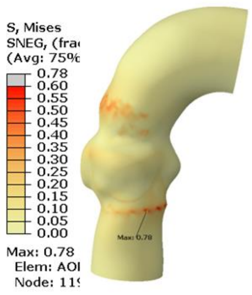	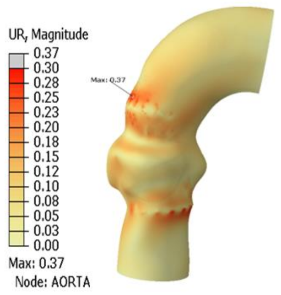
